# Impact on Disease Development, Genomic Location and Biological Function of Copy Number Alterations in Non-Small Cell Lung Cancer

**DOI:** 10.1371/journal.pone.0022961

**Published:** 2011-08-02

**Authors:** Yen-Tsung Huang, Xihong Lin, Lucian R. Chirieac, Ray McGovern, John C. Wain, Rebecca S. Heist, Vidar Skaug, Shanbeh Zienolddiny, Aage Haugen, Li Su, David C. Christiani

**Affiliations:** 1 Department of Epidemiology, Harvard School of Public Health, Boston, Massachusetts, United States of America; 2 Department of Biostatistics, Harvard School of Public Health, Boston, Massachusetts, United States of America; 3 Department of Pathology, Brigham and Women's Hospital, Massachusetts General Hospital, Boston, Massachusetts, United States of America; 4 Cancer Center, Massachusetts General Hospital, Boston, Massachusetts, United States of America; 5 Thoracic Surgery Unit, Massachusetts General Hospital, Boston, Massachusetts, United States of America; 6 Department of Environmental Health, Harvard School of Public Health, Boston, Massachusetts, United States of America; 7 Department of Biological and Chemical Working Environment, National Institute of Occupational Health, Oslo, Norway; 8 Pulmonary and Critical Care Unit, Massachusetts General Hospital, Boston, Massachusetts, United States of America; National Taiwan University Hospital, Taiwan

## Abstract

Lung cancer, of which more than 80% is non-small cell, is the leading cause of cancer-related death in the United States. Copy number alterations (CNAs) in lung cancer have been shown to be *positionally* clustered in certain genomic regions. However, it remains unclear whether genes with copy number changes are *functionally* clustered. Using a dense single nucleotide polymorphism array, we performed genome-wide copy number analyses of a large collection of non-small cell lung tumors (n = 301). We proposed a formal statistical test for CNAs between different groups (e.g., non-involved lung vs. tumors, early vs. late stage tumors). We also customized the gene set enrichment analysis (GSEA) algorithm to investigate the overrepresentation of genes with CNAs in predefined biological pathways and gene sets (i.e., *functional* clustering). We found that CNAs events increase substantially from germline, early stage to late stage tumor. In addition to genomic position, CNAs tend to occur away from the gene locations, especially in germline, non-involved tissue and early stage tumors. Such tendency decreases from germline to early stage and then to late stage tumors, suggesting a relaxation of selection during tumor progression. Furthermore, genes with CNAs in non-small cell lung tumors were enriched in certain gene sets and biological pathways that play crucial roles in oncogenesis and cancer progression, demonstrating the functional aspect of CNAs in the context of biological pathways that were overlooked previously. We conclude that CNAs increase with disease progression and CNAs are both *positionally* and *functionally* clustered. The potential functional capabilities acquired via CNAs may be sufficient for normal cells to transform into malignant cells.

## Introduction

Lung cancer, of which more than 80% is non-small cell type (NSCLC), is the second most common cancer and the leading cause of cancer-related death in the United States [1]. It has been shown in previous studies that NSCLC tumor has more genomic alterations in specific region of the chromosomes, including the copy number gains of partial or whole chromosomal arms on 1q, 3q, 5p and 8q, and the copy losses on 3p, 6q, 8p, 9p, 13q and 17q [2], [3]. That is, copy number alterations in lung cancer do not occur randomly in the genome but are *positionally* clustered. However, where the non-randomness comes from, and furthermore, whether the genes with copy number changes are also *functionally* clustered remains unclear. The goal of this study is to characterize the genome-wide copy number profiles in non-small cell lung cancer both *positionally* and *functionally*.

We have collected 301 NSCLC tumor samples along with 63 paired blood samples and paired adjacent 50 normal tissue samples. Among the tumor samples, a subset of them are late-stage (n = 25). With the heterogeneity of those samples, we are able to establish a genomic model of disease development from germline genome (blood) or pre-cancerous genome (adjacent non-involved tissue), to early stage tumor genome and then to late stage tumor genome. This model also enables us to study the trends in the genome-wide copy number alterations (CNAs) pattern and its selection effects. In addition to focusing on the CNAs profile in tumor samples like previous studies, here we further investigated the difference of CNAs in non-involved tissue, early stage and late stage as well as between adenocarcinoma and squamous cell carcinoma. In order to perform a formal statistical test of genome-wide CNAs pattern between different groups, we proposed a permutation-based global test, in which multiple comparisons, correlation of copy numbers and location of probe loci are fully adjusted.

Gene set enrichment analysis (GSEA) was originally developed for analyses of expression arrays and was used to identify the over-representation of genes belonging to a particular biological category that are associated with biological phenotypes (e.g., stage, histology) [4]. Molecular Signature Database (MSigDB) is a collection of curated gene sets for use with GSEA. Here we show that with modification of the permutation scheme, GSEA can be adapted to explore the over-representation of genes with CNAs on pre-defined MSigDB gene sets (i.e., “*functional* clustering”).

In the “chromosomal theory of cancer”, tumorigenesis is initiated by aneuploidies [5], [6]. For tumorigenesis, six necessary acquired capabilities have been proposed: self-sufficiency in growth signals, insensitivity to anti-growth signals, evading apoptosis, limitless replicative potential, sustained angiogenesis and tissue invasion and metastasis [7]. Since we hypothesize that there is functional clustering of genes with CNAs, we sought to investigate whether CNAs are a sufficient mechanistic strategy to acquire the above capabilities; that is, whether the functional clustering of genes with CNAs provides supporting evidence for the chromosomal theory of cancer.

## Materials and Methods

### Ethics Statement

Written informed consent was obtained from all patients. The study was approved by the institutional review boards of MGH, the Harvard School of Public Health, and the Norwegian Data Inspectorate, and The Local Regional Committee for Medical Research.

### Study population and specimens

A series of 301 snap-frozen tumor samples from NSCLC patients was collected during surgery or biopsy from the Massachusetts General Hospital (MGH), Boston, MA and the National Institute of Occupational Health, Oslo, Norway. We also included 50 additional specimens of paired non-neoplastic lung parenchyma from the Norwegian patients and 63 paired blood samples from the MGH patients, all of which were used as the reference group of copy number estimation.

### DNA quality, histopathology and genechip

DNA samples were extracted from tumor and non-neoplastic lung parenchyma after manual microdissection of 5-μ histopathologic sections. For DNAs from MGH patients, a pathologist (L.R.C.) who had no knowledge of the clinical and genetic information reviewed all sections for each patient. Each specimen was evaluated for amount and quality of tumor cells and histologically classified using the WHO criteria. The Norwegian specimens were all resected collected and prepared in the same way. Specimens with lower than 70% cancer cellularity, inadequate DNA concentration (<50 ng/µL), or a smearing pattern in gel electrophoresis were not included for genotyping. A total of 414 DNA samples (301 from tumors, 63 from paired blood samples and 50 from paired non-involved lung samples) were hybridized onto Affymetrix 250K Nsp GeneChip®, which contains 262,264 probes (256,554 probes on somatic chromosomes and 5,710 probes on sex chromosome).

### Data preprocessing

Copy numbers were obtained with dChip software [8]. The probe intensities were calculated by model-based expression after invariant set normalization. For each SNP in each sample, the raw copy number was computed as signal×2÷(mean signal of reference samples at this SNP) using blood and non-neoplastic tissue samples as the referent. Inferred copy numbers were computed from the raw copy numbers by median smoothing with the window of 11 SNPs for each locus of 262,264 SNPs. Only 256,554 probes on somatic chromosomes were analyzed. The SNP probes were mapped to the RefSeq genes with 2 kb extension both upstream and downstream using the UCSC Genome Browser. Among the 256,554 probes on somatic chromosomes, 104,256 probes were mapped to 11,700 genes.

### Statistical analysis

Copy number gains and losses were analyzed separately. Copy number gains were defined as inferred copy numbers (CN) ≥2.7 and copy number losses were defined as inferred copy numbers ≤1.3. The cut-offs were chosen to detect copy number ≥3 and ≤1 by tolerating 30% normal tissue contamination. Note that 70% cancer cellularity was the threshold for our pathological check of quality. The prevalence of the subjects with CNAs was plotted across the genome. For each locus, the numbers of patients having CNAs were assumed to follow a binomial distribution with the sample size as the total number of subjects and the null probability estimated empirically from the data: total probes with CN≥2.7 (or CN≤1.3)÷(256,554×sample size). Significance in genome-wide copy number alterations was determined by calculating the exact p values for each of the 256,554 loci, and q values were calculated to control for multiple comparisons across the genome using the false discovery rate [9], [10]. For each gene mapped by multiple probes, the probe with the highest proportion of samples having CNAs, or equivalently, the smallest p value was chosen to represent the CNAs feature of the gene.

Here we proposed a permutation-based global test for the genome-wide CNAs patterns between two groups were different, we applied two-sample tests for binomial data by calculating the standardized difference of two proportions for each locus as: 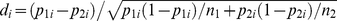 where *p_ji_* is the estimated proportion (stabilized by adding 0.5 in the numerator) of CN gains (or losses) for group *j* at locus *i* and *n_j_* is the sample size in group *j*. We summed up *d_i_* ^2^ over *i* across the 256,554 loci to calculate the observed total standardized squared difference (*D_observed_*) across the genome. By permuting the two groups and carrying out the above procedure for 10,000 times, we obtained a non-parametric null distribution (***D*** *_null_*). Then p values were obtained by comparing *D_observed_* and ***D*** *_null_*. The advantage of this proposed test is that it provides a valid global test for the overall genome-wide difference by accounting for multiple comparisons and correlation of CNAs between different loci.

**Figure 1:**
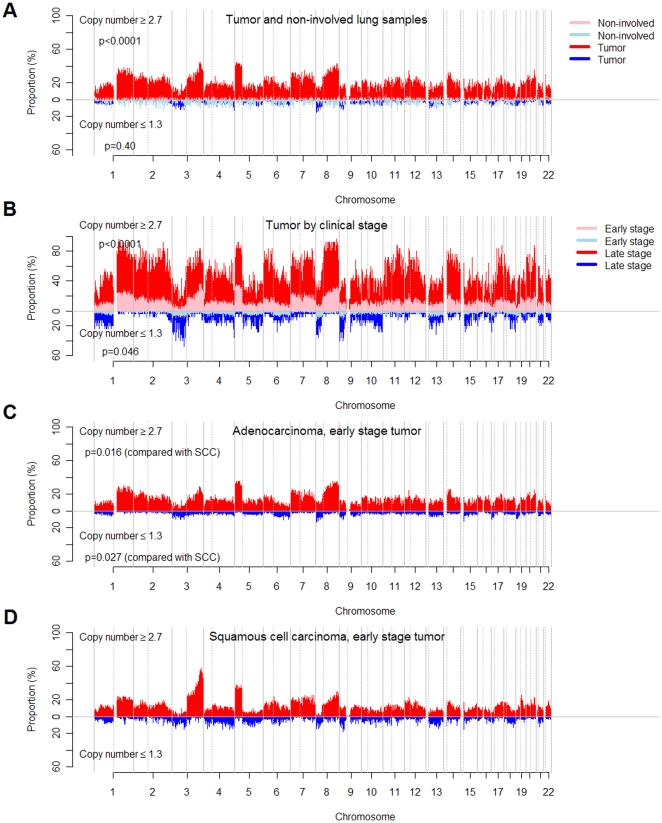
Genome-wide copy number alterations in blood, non-involved lung tissue and tumor of NSCLC patients. The x axis represents genomic locations, which were ordered by the somatic chromosomes. The y axis represents the prevalence (%) of NSCLC patients having copy number ≥2.7 (red or pink) and ≤1.3 (blue or light blue) in non-involved lung tissue and total tumor (A), early stage tumor and late stage tumor (B), early stage tumor of adenocarcinoma (C) and early stage tumor of squamous cell carcinoma (SCC) (D). The corresponding plots of −log_10_(q values) were shown in Fig. S1. The p values of comparing genome-wide CNAs patterns between non-involved tissue samples and total tumors are <0.0001 for gains and 0.40 for losses by the permutation-based global test with details described in Methods. The p values comparing early stage and late stage tumors are <0.0001 for gains and 0.046 for losses; the p values comparing early stage adenocarcinoma (C) and early stage squamous cell carcinoma (D) are 0.016 for gains and 0.027 for losses.

Using the global test described above, we tested the genome-wide CNAs patterns between blood and tumors, non-involved lung and tumors, early stage and late stage tumors, early stage adenocarcinoma and squamous cell carcinoma tumors (Figure 1). To further confirm the results, we performed the following matched analyses. Since the blood and non-involved lung samples were paired to subset of tumor samples, we can compare the difference of genome-wide CNAs restricted to those with available samples on blood and tumors or on non-involved and tumors. For each late stage tumor, we selected one corresponding early stage tumor sample with closest smoking pack-years. The distribution of gender, histology and smoking pack-years showed no significant difference in the matched early and late stage tumors. The matched analyses showed similar results to those in Figure 1. (Figure S2)

Both the total probes (TP) and the probes locating within genes (GP) in which CNAs were detected were calculated for each individual. Comparison of TP and GP across different subgroups allows studying the pattern of selection of genomic regions where CNAs occur, under the assumption that the probes on the chip were chosen randomly without considering linkage disequilibrium. The ratio of GP vs. TP (termed as G/T ratio) was calculated to estimate the selection of CNAs with respect to the gene location. Under the null hypothesis that CNAs occur randomly relative to where genes locate, we would expect the null ratio of 104,256/256,554 = 40.64%, where 104,256 is the number of probes located within genes on the chip. By comparing the G/T ratios to the null ratio 40.64%, we were able to test whether CNAs occurred preferentially away from genes. Comparing G/T in different subgroups (e.g., germline vs. tumor) enabled us to investigate the magnitude of this preferential selection among different groups. The comparisons of TP, GP or G/T ratios between two groups were performed using unpaired two-sided student t test assuming unequal variances.

Gene set analyses were performed using the modified Gene Set Enrichment Analysis (GSEA) algorithm. GSEA was originally proposed for gene expressions between groups [4]. Since we did not attempt to associate the CNAs with other covariates but simply investigate the enrichment of CNAs in a single group, we modified the algorithm regarding the generation of null distribution of the enrichment score. We are interested in whether CNAs in a gene set are significantly higher than other gene sets. Instead of permuting the group label, we permuted the gene labels for 20,000 times to create the null distribution. The discovery (n = 151) and validation sets (n = 150) randomly picked from the 301 tumors were similar in many demographic and clinical characteristics. (Table S1) Primary analysis was done using the discovery data and validation was performed using the validation dataset. Only gene sets that were significant (p<0.05) in both sets were reported. The gene sets analyzed in this study were taken from the Molecular Signatures Database (MSigDB) of the Harvard/MIT Broad Institute, including gene families, curated gene sets and gene ontology gene sets. Only 1619 gene sets with at least 15 gene members in our data were analyzed to achieve robustness.

## Results

### CNAs and disease development

**Figure 2:**
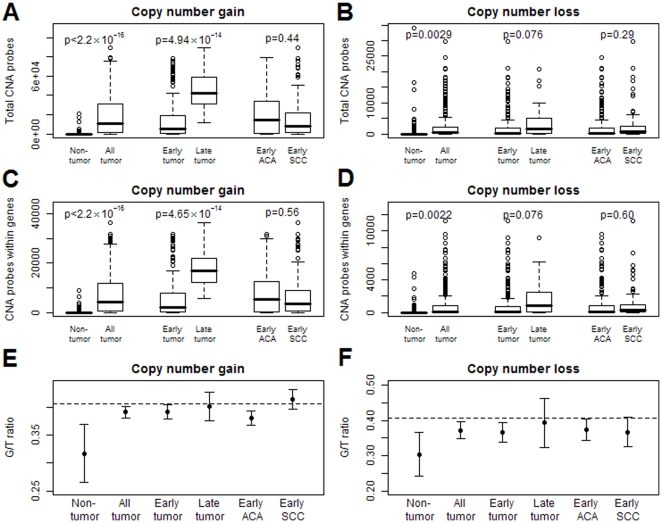
Total probes (TP) and probes locating within genes (GP) in which CNAs were detected, and the mean of G/T ratios. A, B, Counts of the total probes (TP) where CNAs events (A: copy number gains, B: copy number losses) occur were plotted for blood and non-involved tissue, total tumor, early stage tumor, late stage tumor, early stage adenocarcinoma (ACA) and early stage squamous cell carcinoma (SCC). C, D, Counts of the probes within genes (GP) in which CNAs events (C: copy number gains, D: copy number losses) were detected in the same six subgroups. E, F, Mean and its 95% confidence interval of G/T ratios in the six subgroups for copy number gains (E) and losses (F); and the dashed lines represent the null G/T ratio on the chip (104,256/256,554 = 40.64%). Non-tumor: blood (n = 63) and non-involved tissue (n = 50); All tumor: total 301 NSCLC tumors; Early tumor: stage I and II NSCLC tumors (n = 246); Late tumor: stage III and IV NSCLC tumors (n = 25); Early ACA: early stage adenocarcinoma tumors (n = 208); Early SCC: early stage squamous cell carcinoma tumors (n = 93).

A series of 301 tumor samples was collected from NSCLC patients, the characteristics of which are shown in Table S1. The genome of blood or non-involved lung tissue had substantially fewer CNAs events than did the tumor genome, especially in copy number gains (losses: p = 0.038 in blood vs. tumor, p = 0.40 in non-involved tissue vs. tumor; gains: p<0.0001 in both) (Figure 1A). The false discovery rates (q values) of the 256,554 loci for blood, non-involved tissue, tumors (in total, by clinical stage or by histology) are shown in Figure S1. There were substantial CNAs on chromosomes 3, 5 and 8, illustrated in Figures S3, S4, and S5. Because Affymetrix® 250K Nsp GeneChip probes were selected randomly across the genome, it is reasonable to assume that the number of the probes that detect copy number alterations is proportional to the genomic span of CNAs events. The average number of probes that detect copy number gains was 718 in blood and non-involved tissue, which was much lower than the 19,469 in tumor (p<2.20×10^−16^) (Figure 2A). The pattern was also found in copy number losses (950 vs. 2,586, p = 0.0029) (Figure 2B). Furthermore, there are more copy number gains than copy number losses in tumors (p<2.20×10^−16^), which suggests that the copy number losses are more deleterious [11].

The prevalence of CNAs events among NSCLC patients was associated with clinical stage, especially in amplification. The proportion of patients with copy number alterations in late stage tumor was more than twice of that in early stage. (Figure 1B) Between the two groups, we performed the global tests for the paired difference of the proportion of CNAs for each locus across the genome, and showed highly significant difference in gains (<0.0001) and a marginally significant difference in losses (p = 0.046) after accounting for multiple comparisons. Similarly, the average number of probes that detect copy number gains was 14,029 in early stage and 45,792 in late stage (p = 4.94×10^−14^) (Figure 2A). For copy number losses, they were 2,419 and 4,395, respectively (p = 0.076) (Figure 2B). Excluding those with adjuvant chemo- or radio-therapy still preserved the significant trend and the corresponding numbers (p value) were 8,501 and 41,608 (p = 5.43×10^−5^) in gains and 2,099 and 5,947 (p = 0.017). Adenocarcinoma and squamous cell carcinoma subtypes show a significant difference in testing paired proportion (Figure 1C and 1D) (p = 0.016 in gains and p = 0.027 in losses), but no difference in total CNAs events (Figure 2A and 2B) (p = 0.44 in gains and p = 0.29 in losses), indicating that the genome-wide CNAs patterns of the two cell types may be different even though the numbers of total events are similar.

### CNAs selection of gene location and disease development

By calculating the G/T ratio (see Materials and Methods), we investigated the selection of CNAs with respect to gene locations during cancer development. In blood or non-involved tissues, the G/T ratios were lower than the null (40.64%) in gains (31.71%, p = 0.00098) and losses (30.38%, p = 0.0014) (Figure 2E and 2F), which indicates that CNAs events are more likely to happen outside genes in the germline as a result of natural selection. In tumor genome, the selection effect still exists even though it has been relaxed to certain extent. That is, the G/T ratios in tumors were significantly higher than those in germline (p = 3.28×10^−5^ in gains, p = 0.015 in losses), but they were still significantly lower than the null ratio (39.16%, p = 0.0068 in gains; 37.17%, p = 0.0052 in losses). However, such a selection effect was not observed in late stage tumors, i.e., CNAs events have a similar chance to occur within and outside genes.

### CNAs in oncogenes and tumor suppressor genes

104,256 (40.64%) out of 256,554 probes of somatic chromosomes on the chip were mapped to 11,700 genes with 2 kb extension both upstream and downstream to include promoter and flanking regions. There were 32 known oncogenes in which >10% of patients had copy number gains (Table 1) and 16 tumor suppressor genes in which >1% patients had copy number losses (Table 2). We also identified 45 genes (including oncogenes and non-oncogenes) with >35% (p≤1.50×10^−42^) having copy number amplifications (Table S2) and 9 genes (*CSMD1*, *SGCZ*, *PDZRN3*, *NISCH*, *CACNA2D3*, *UBE2E2*, *MCPH1*, *PHF7* and *DOCK5*) with >10% (p≤1.53×10^−21^) having copy number deletions.

**Table 1:** Thirty two oncogenes with >10% NSCLC patients having copy number gains.

	Symbol	Full name of oncogene	Position	% of patients with CN gains	P value*
1	*TRIO*	Triple functional domain	5p15.2	38.21	3.30×10^−50^
2	*EGFR*	epidermal growth factor receptor	7p12	26.58	2.26×10^−23^
3	*ERBB4*	v-erb-a erythroblastic leukemia viral oncogene homolog 4	2q33.3–34	26.58	2.26×10^−23^
4	*RUNX1T1*	Runt-related transcription factor 1	8q22	26.25	9.97×10^−23^
5	*ABL2*	v-abl Abelson murine leukemia viral oncogene homolog 2	1q24–25	23.59	7.66×10^−18^
6	*ETV5*	ets variant 5	3q28	22.92	1.07×10^−16^
7	*ETV1*	ets variant 1	7p21.3	22.26	1.38×10^−15^
8	*A2BP1*	ataxin 2-binding protein 1	16p13.3	22.26	1.38×10^−15^
9	*MYC*	v-myc myelocytomatosis viral oncogene homolog (avian)	8q24.21	21.59	1.66×10^−14^
10	*TERT*	telomerase reverse transcriptase	5p15.33	20.60	5.97×10^−13^
11	*AKT3*	v-akt murine thymoma viral oncogene homolog 3	1q43–44	20.60	5.97×10^−13^
12	*KRAS*	v-Ki-ras2 Kirsten rat sarcoma viral oncogene homolog	12p12.1	20.27	1.90×10^−12^
13	*ETV6*	ets variant 6	12p13	19.93	5.90×10^−12^
14	*PTPN1*	protein tyrosine phosphatase, non-receptor type 1	20q13.1–13.2	19.27	5.37×10^−11^
15	*PIK3CA*	phosphoinositide-3-kinase, catalytic, alpha polypeptide	3q26.3	18.60	4.50×10^−10^
16	*PGR*	progesterone receptor	11q22–23	16.61	1.58×10^−7^
17	*BRAF*	v-raf murine sarcoma viral oncogene homolog B1	7q34	15.95	9.28×10^−7^
18	*ERG*	v-ets erythroblastosis virus E26 oncogene homolog (avian)	21q22.3	15.61	2.17×10^−6^
19	*CSMD1*	CUB and Sushi multiple domains 1	8p23.2	15.28	4.97×10^−6^
20	*ELF5*	E74-like factor 5 (ets domain transcription factor)	11p13–12	14.95	1.11×10^−5^
21	*PDE4D*	phosphodiesterase 4D	5q12	14.62	2.41×10^−5^
22	*FGFR1*	fibroblast growth factor receptor 1	8p11.2–11.1	14.62	2.41×10^−5^
23	*EPHA3*	EPH receptor A3	3p11.2	14.62	2.41×10^−5^
24	*CDK6*	cyclin-dependent kinase 6	7q21–q22	14.62	2.41×10^−5^
25	*ESR2*	Estrogen receptor 2	14q23.2	14.62	2.41×10^−5^
26	*JAK2*	Janus kinase 2	9p24	14.62	2.41×10^−5^
27	*WWOX*	WW domain containing oxidoreductase	16q23.3–24.1	13.95	0.000106
28	*PDGFRA*	platelet-derived growth factor receptor, alpha polypeptide	4q11–13	13.95	0.000106
29	*CCND2*	cyclin D2	12p13	13.95	0.000106
30	*CCNE1*	cyclin E1	19q12	13.62	0.000214
31	*RBL1*	retinoblastoma-like 1	20q11.2	11.30	0.0136
32	*MET*	met proto-oncogene (hepatocyte growth factor receptor)	7q31	11.30	0.0136

*P values were calculated to test the significance of observing the percentage (%) of patients with copy number gains (copy number ≥2.7) on the oncogenes, given that the null probability of copy number gains is 0.076, which is empirically estimated from the data.

**Table 2:** Sixteen tumor suppressor genes with >1% NSCLC patients having copy number losses.

	Symbol	Full name of tumor suppressor gene	Position	% of patients with copy number losses	P value*
1	*CDKN2A*	cyclin-dependent kinase inhibitor 2A	9p12	9.63	1.87×10^−19^
2	*FHIT*	Fragile histidine triad gene	3p14.2	6.64	6.33×10^−11^
3	*BRCA2*	breast cancer 2, early onset	13q12.3	4.65	3.2×10^−6^
4	*TGFBR2*	transforming growth factor, beta receptor II	3p22	4.31	1.55×10^−5^
5	*FLCN*	Folliculin	17p11.2	3.65	0.00029
6	*RB1*	retinoblastoma 1	13q14.2	2.66	0.012
7	*NF2*	neurofibromin 2 (merlin)	22q12.2	2.66	0.012
8	*PTEN*	phosphatase and tensin homolog	10q23.3	1.66	0.19
9	*EP300*	E1A binding protein p300	22q13.2	1.66	0.19
10	*FBXW7*	F-box and WD repeat domain containing 7	4q31.3	1.66	0.19
11	*APC*	adenomatous polyposis coli	5q21–q22	1.66	0.19
12	*NOTCH1*	Notch homolog 1, translocation-associated	9q34.3	1.66	0.19
13	*FAT3*	FAT tumor suppressor homolog 3	11q14.3	1.33	0.36
14	*SMARCB1*	SWI/SNF related, matrix associated, actin dependent regulator of chromatin, subfamily b, member 1	22q11	1.33	0.36
15	*SMAD4*	mothers against decapentaplegic homolog 4	18q21.1	1.33	0.36
16	*SMAD2*	mothers against decapentaplegic homolog 2	18q21.1	1.33	0.36

*P values were calculated to test the significance of observing the percentage (%) of patients with copy number losses (copy number ≤1.3) on the tumor suppressor genes, given that the null probability of copy number losses is 0.010, which is empirically estimated from the data.

### Gene sets enriched with CNAs genes

Since the genes with CNAs were under selection, we hypothesized that these genes should be involved in similar biologic functions, which subsequently favor fitness of cells during tumorigenesis and/or cancer cell proliferation. Therefore, we investigated further whether genes with CNAs were enriched in 1619 predefined gene sets. To avoid false positive findings when testing 1619 gene sets, the analyses were done with a discovery-and-validation process. In the discovery set, the genes with copy number amplifications were significantly enriched in 152 gene sets (p<0.05); 119 of them were validated in the validation set at significance level of 0.05. For copy number deletions, 109 gene sets were found in the discovery set (p<0.05) and 52 were validated. We also investigated the 119 and 52 validated gene sets in early stage and late stage tumors and only those significantly enriched in both subgroups are reported (89 in gains and 27 in losses; Tables S3 and S4) We present 26 gene sets with particular relevance to tumor biology that have enrichment of copy number gains or losses in our samples in Tables 3 and 4 and the corresponding gene set enrichment plots in Figures S6 and S7. Further investigating the gene set enrichment in the blood and non-involved lung tissue, we found many of the 89 and 27 validated gene sets were also enriched in the genome of non-involved lung tissue, including gains in G protein signaling pathway, *EDG1* pathway, integrin-mediated cell migration pathway and losses in regulations of autophagy and mitotic cell cycle. (Tables S3 and S4)

**Table 3:** P values of the selective sixteen out of eighty-nine pathways and gene sets with enrichment of genes with copy number gains (the complete eighty-nine gene sets are listed in Table S3).

	MSigDB ID of gene set: brief description	Blood	Non-involved lung	Discovery set	Validation set	Early stage tumors	Late stage tumors	All tumors
1	AGUIRRE_PANCREAS_CHR8: Genes on chromosome 8 with copy-number-driven expression in pancreatic adenocarcinoma.	0.46	<5×10^−5^	<5×10^−5^	<5×10^−5^	<5×10^−5^	5×10^−5^	<5×10^−5^
2	BRCA_PROGNOSIS_NEG: Genes whose expression is consistently negatively correlated with breast cancer outcomes - higher expression is associated with metastasis and poor prognosis	0.64	<5×10^−5^	<5×10^−5^	<5×10^−5^	<5×10^−5^	<5×10^−5^	<5×10^−5^
3	SMITH_HCV_INDUCED_HCC_UP: Genes highly expressed in hepatitis C-related hepatocellular carcinoma	0.57	<5×10^−5^	0.00025	5×10^−5^	<5×10^−5^	0.00085	5×10^−5^
4	HSA04514_CELL_ADHESION_MOLECULES: Genes involved in cell adhesion molecules (CAMs)	0.029	0.39	0.00085	0.00040	0.0049	0.00025	0.00040
5	ONCOGENE: Census of human cancer genes	0.37	0.51	0.0011	0.0047	0.0018	0.015	0.00065
6	G_PROTEIN_SIGNALING: Genes involved in G protein signaling	0.41	<5×10^−5^	0.0016	0.0021	0.0030	0.011	0.0010
7	HSA04610_COMPLEMENT_AND_COAGULATION_CASCADES: Genes involved in complement and coagulation cascades	0.14	<5×10^−5^	0.0012	0.0028	0.00065	0.0036	0.00145
8	CELL_ADHESION: The attachment of a cell, either to another cell or to the extracellular matrix, via cell adhesion molecules.	0.00050	0.76	0.0026	0.0041	0.012	0.0012	0.0024
9	AT1RPATHWAY: Binding of angiotensin II to *AT1-R* activates Ca2+ signaling and the *JNK* pathway.	0.00050	<5×10^−5^	0.0041	0.0038	0.0068	0.016	0.0031
10	AGUIRRE_PANCREAS_CHR7: Genes on chromosome 7 with copy-number-driven expression in pancreatic adenocarcinoma.	0.93	<5×10^−5^	0.018	0.00025	0.0026	0.0019	0.0033
11	EDG1PATHWAY: The lipid *S1P* is an *EDG1* ligand promoting chemotaxis via *Rac1* and cell survival and proliferation via *ERK* activation.	0.031	<5×10^−5^	0.015	0.0089	0.010	0.022	0.0085
12	LOTEM_LEUKEMIA_UP: Genes upregulated in myeloid leukemia and normally expressed in other bodily tissues.	0.62	<5×10^−5^	0.0048	0.033	0.0077	0.041	0.0089
13	BRCA_ER_NEG: Genes whose expression is consistently negatively correlated with estrogen receptor status in breast cancer - higher expression is associated with *ER*-negative tumors	0.84	0.94	0.0069	0.0099	0.0037	0.012	0.0090
14	MCALPAINPATHWAY: In integrin-mediated cell migration, calpains digest links between the actin cytoskeleton and focal adhesion proteins.	0.80	<5×10^−5^	0.019	0.0080	0.0078	0.047	0.010
15	HSA05211_RENAL_CELL_CARCINOMA: Genes involved in renal cell carcinoma	0.32	0.32	0.023	0.031	0.0082	0.025	0.020
16	WNT_SIGNALING: *Wnt* signaling genes	0.17	0.31	0.020	0.025	0.021	0.0077	0.024

**Table 4:** P values of the selective ten out of twenty seven pathways and gene sets with enrichment of genes with copy number losses (the complete twenty-seven gene sets are listed in Table S4).

	MSigDB ID of gene set: brief description	Blood	Non-involved lung	Discovery set	Validation set	Early stage tumors	Late stage tumors	All tumors
1	AGUIRRE_PANCREAS_CHR9: Genes on chromosome 9 with copy-number-driven expression in pancreatic adenocarcinoma.	0.25	0.57	<5×10^−5^	5×10^−5^	<5×10^−5^	<5×10^−5^	<5×10^−5^
2	HSA04080_NEUROACTIVE_LIGAND _RECEPTOR_INTERACTION: Genes involved in neuroactive ligand-receptor interaction	0.061	0.00050	0.00070	<5×10^−5^	<5×10^−5^	0.0042	5×10^−5^
3	CELL_CELL_SIGNALING: Genes annotated by the GO term GO:0007267. Any process that mediates the transfer of information from one cell to another.	0.80	0.061	0.0015	0.013	0.0017	0.0030	0.00070
4	HSA04140_REGULATION_OF_AUTOPHAGY: Genes involved in regulation of autophagy	0.16	<5×10^−5^	0.00065	0.0046	0.00095	0.00010	0.00085
5	SIGNAL_TRANSDUCTION: Genes involved in signal transduction	0.21	0.23	0.012	0.0019	0.011	0.022	0.0015
6	CELL_SURFACE_RECEPTOR_LINKED_SIGNAL_TRANSDUCTION_GO_0007166: Genes annotated by the GO term GO:0007166. Any series of molecular signals initiated by the binding of an extracellular ligand to a receptor on the surface of the target cell.	0.35	0.034	0.021	0.0017	0.027	0.00075	0.0031
7	CELL_CELL_ADHESION: Genes annotated by the GO term GO:0016337. The attachment of one cell to another cell via adhesion molecules.	0.53	0.069	0.0071	0.0085	0.0028	0.00035	0.0039
8	HDACI_COLON_CUR24HRS_DN: Downregulated by curcumin at 24 hrs in SW260 colon carcinoma cells	0.92	<5×10^−5^	0.0070	0.0020	0.0094	0.037	0.0050
9	REGULATION_OF_MITOTIC_CELL_CYCLE: Genes annotated by the GO term GO:0007346. Any process that modulates the rate or extent of progress through the mitotic cell cycle.	0.64	<5×10^−5^	0.0010	0.049	0.0075	0.017	0.0053
10	CHROMATIN_MODIFICATION: Genes annotated by the GO term GO:0016568. The alteration of DNA or protein in chromatin, which may result in changing the chromatin structure.	0.98	0.71	0.019	0.022	0.049	0.0095	0.021

## Discussion

The genome-wide CNAs pattern from our analyses is similar to those published in previous literatures [2], [3], [12]. Many of the oncogenes with copy number amplifications reported here is also consistent with previous studies [13], [14], [15], [16]. The major strength of this study is its large sample size, availability of paired blood and non-involved tissue samples and detailed demographic/clinical information, discovery-validation process and the novel statistical analyses. The proposed global test for genome-wide CNAs provide us the opportunities to test CNAs difference by simultaneously taking the genomic locations, correlation of copy numbers and multiple comparisons into account. The customized GSEA for CNAs, on the other hand, can serve as a useful tool to analyze the genome-wide copy numbers in the functional and biological context, linking the sophisticated CNAs data to the knowledge of gene categories, biological pathways and previous studies. There are still limitations in our study. First, blood and non-involved tissue samples can be obtained from only subset of the 301 patients. Second, we are unable to collect the CNAs data from normal subjects or patients without lung cancer, which may provide us a better insight into how the genome-wide CNAs profile in NSCLC patients differs from that in normal subjects or non-cancer patients. Thirdly, even though the gene sets analyses can serve to formulate biological hypotheses, further investigation to study the roles of gene sets/pathways with CNAs in tumorigenesis is required.

The DNA materials analyzed in this study all come from NSCLC patients, so the genome of blood and non-involved tissue may not be viewed as a normal genome. We use DNAs from blood, non-involved lung tissue, early-stage tumor and late-stage tumor to represent the sequential stages of cancer development and progression. We discovered that copy number alterations increase with cancer development, but that selection with respect to gene location decreases. That is, there is a monotonic increase in copy number alterations from blood and non-involved lung tissue, to early stage and then to late stage tumors. (Figures 2A and 2B) On the other hand, copy number changes tend to occur *away* from gene location in blood or non-involved tissue, but this trend decreases in tumors, especially in late stage. (Figures 2E and 2F) The increase of CNAs reflects the accumulation of somatic copy number changes due to genomic instability, in which cellular hypoxic stress in cancer might play a key role via perturbation of DNA replication and replication of non-contiguous DNA segments [17], [18].

Similarly, we expect to see the accumulation of the number of gene sets hit by CNAs from blood, non-involved tissue and then to tumor, rather than the abrupt occurrence in tumor. Out of our reported 89 gene sets of copy number gains, the numbers of significant gene sets are 7 in blood, 46 in non-involved tissue, 89 in tumors. (Table S3) Out of the reported 27 gene sets of copy number losses, the numbers are 2 in blood, 8 in non-involved tissue, 27 in tumors. (Table S4)

Selection of CNAs with respect to gene location during evolution is also reported in *Drosophila* [11]. Here we show a similar selection effect in the tumor genome even though it is relaxed to some extent. We hypothesize that the selection in tumor occurs during early cancer development on top of the consequence of evolutionary selection as reflected in germline. These findings illustrate that the purifying selection occurring in the germline as the result of species evolution may also occur in tumor as a selecting effect during oncogenesis and tumor progression. We also hypothesize that the biological pathways with CNAs we observe in Tables S3 and S4 are the consequence of such selection. That is, the large-scale alterations may hit many different gene and biological pathways randomly, but only the cells acquiring the growth advantage via CNAs (e.g., amplified oncogene signaling pathway or deleted tumor suppressor gene pathway) will survive and become dominant. The finding that CNAs tend to occur away from the gene also reflects the non-randomness of CNAs occurrence.

Oncogenes can mimic normal growth signaling such that the cancer cell reduces the dependence on exogenous growth stimulation. Thus, the amplification of oncogenes is an essential step in tumorigenesis. In lung cancer, *KRAS*, *MYC*, *EGFR*, and *ERBB* family are well-known oncogenes [19], [20], [21], [22], and all of them were found to be highly significant in copy number gains. Furthermore, genes with copy number amplifications were also over-represented in the oncogenes as a category defined by the census of human cancer genes [23]. This finding suggests that oncogenesis may also result from different sets of oncogenes in addition to the above well-known ones.

Genes with CNAs in NSCLC are also found to be highly associated with genes involved in other cancers, including liver, breast, kidney and pancreatic cancers. We found that genes with copy number gains in NSCLC are more likely to be the highly expressed genes in hepatitis C-related hepatocellular carcinoma [24] (p = 0.00005) and renal cell carcinoma [25], [26] (p = 0.020). Genes with copy number amplifications are also significantly enriched in the genes of poor prognosis signature of breast cancer [27] (p<0.00005), which may explain the poorer prognosis of lung cancer than breast cancer. Moreover, our analysis showed the genes with copy number gains in lung were enriched in the genes on chromosomes 7 and 8, shown to have copy-number-driven expression in pancreatic adenocarcinoma [28] (p = 0.0033 and <0.00005, respectively), and those with copy number losses were enriched in the genes with CNAs on chromosome 9, also associated with gene expression in pancreatic tumor (p<0.00005). This suggests that the gene dosage, as it were, of genes with CNAs in NSCLC may also be positively related to expression level. These results also suggest that cancer cells emerging from different tissue origins share similar machinery for oncogenesis and tumor invasion.

Our gene set analysis suggests that cells may acquire the common capacities of tumor [7] through CNAs: gains in oncogenes (self-sufficiency in growth signals; evading apoptosis), gains in *Calpain* pathway (insensitivity to anti-growth signals; tissue invasion and metastasis, sustained angiogenesis), gains in *EDG1* pathway (evading apoptosis and self-sufficiency in growth signals), gains in *ERBB* signaling (self-sufficiency in growth signals), gains in *WNT* signaling (self-sufficiency in growth signals), losses in regulation of autophagy (evading apoptosis), gains in telomerase reverse transcriptase (*TERT*) (limitless replicative potential), and losses in tight junction and cell adhesions (tissue invasion and metastasis). Such results provide supporting evidence of the ‘chromosomal cancer theory’ [5], [6]: cancer is a disease caused by aneuploidy or large-scale alterations of chromosome. It is because the large-scale duplication or deletion is more likely to alter the copy number of significant numbers of genes in numerous biological pathways. However, our results do not disagree with the notion that duplication/deletion of a gene play a critical role in cancer development. The occurrence of aneuploidy or large-scale alterations requires an environment with genome instability, which is likely to be facilitated by duplication/deletion or mutation of genes critical in DNA repair, recombination and duplication. Thus, they do not exclude the possibilities that CNAs may be initiated by single or small sets of mutated genes and require further investigation.

The finding that CNAs occur preferentially in certain regions and chromosome arms (i.e., *positional* clustering) is consistent with previous studies [2], [3], [12]. Another finding that genes with CNAs tend to involve in certain biological functions and pathways (i.e., *functional* clustering) is novel and can provide a possible explanation to the *positional* clustering: the CNAs hot spots/regions in NSCLC may harbor genes that execute similar biological functions or belong to the crucial pathways. That is, our work is more providing biological insight into the *positional* clustering rather than distinguishing the *functional* clustering from *positional* clustering.

We conclude that CNAs events increase but their selection with respect to gene location decreases from germline to early stage tumors, and then to late stage tumors, and that CNAs in NSCLC tumor are both *positionally* and *functionally* clustered. The functional characteristics of gene sets enriched with CNAs genes provide us better insight into the mechanisms of oncogenesis and cancer progression, and have the potential for improving patient management by drug targeting in the future for this prevalent and devastating disease.

## Supporting Information

Figure S1 Significance of genome-wide copy number alterations in blood, non-involved lung tissue and tumor of NSCLC patients. The x axis represents genomic locations, which were ordered by the somatic chromosomes. The y axis represents −log_10_(q values) of NSCLC patients having copy number ≥2.7 (red) and ≤1.3 (blue) in blood (A), non-involved lung tissue (B), total tumor (C), early stage tumor (D) and late stage tumor (E), early stage tumor of adenocarcinoma (F) and early stage tumor of squamous cell carcinoma (G).
(TIFF)

Figure S2 The comparisons of copy number alterations in paired or matched samples. The proportion of copy numbers ≥2.7 (red and pink) and ≤1.3 (blue and light blue) in paired blood and tumor samples (A), paired non-involved lung and tumor samples (B), and matched early stage and late stage tumors (C). The p values indicate the statistical significance from the global CNAs test between two groups.
(TIFF)

Figure S3 Copy numbers in chromosome 3. The main panel represents the copy numbers of blood, non-involved lung tissue and tumor as indicated. The bottom panel illustrates copy numbers with their corresponding colors (red color indicates copy number gains and blue color indicates copy number losses). The right panel shows the corresponding −log_10_(q values) for each locus on the chromosome; the red and blue colors indicate the −log_10_(q values) for copy number gains and losses, respectively.
(TIFF)

Figure S4 Copy numbers in chromosome 5. The main panel represents the copy numbers of blood, non-involved lung tissue and tumor as indicated. The bottom panel illustrates copy numbers with their corresponding colors (red color indicates copy number gains and blue color indicates copy number losses). The right panel shows the corresponding −log_10_(q values) for each locus on the chromosome; the red and blue colors indicate the −log_10_(q values) for copy number gains and losses, respectively.
(TIFF)

Figure S5 Copy numbers in chromosome 8. The main panel represents the copy numbers of blood, non-involved lung tissue and tumor as indicated. The bottom panel illustrates copy numbers with their corresponding colors (red color indicates copy number gains and blue color indicates copy number losses). The right panel shows the corresponding −log_10_(q values) for each locus on the chromosome; the red and blue colors indicate the −log_10_(q values) for copy number gains and losses, respectively.
(TIFF)

Figure S6 Gene set enrichment plot for the 16 selective gene sets with copy number gains (corresponding to Table 3). For each panel, the top indicates the location of genes from the gene set within the list sorted by the proportion of patients with copy number gains (left to right: higher proportion to lower); the bottom is the plot of running enrichment scores.
(TIFF)

Figure S7 Gene set enrichment plot for the selective 10 gene sets with copy number losses (corresponding to Table 4). For each panel, the top indicates the location of genes from the gene set within the list sorted by the proportion of patients with copy number losses; the bottom is the plot of running enrichment scores.
(TIFF)

Table S1 Characteristics of the discovery and validation tumor samples^*^.
(DOC)

Table S2 Forty-five genes with >35% patients having copy number gains.
(DOC)

Table S3 Eighty-nine pathways and gene sets with enrichment of genes with copy number gains (with p<0.05 in discovery set, validation set, early stage and late stage tumors).
(DOC)

Table S4 Twenty-seven pathways and gene sets with enrichment of genes with copy number losses (with p<0.05 in discovery set, validation set, early stage and late stage tumors).
(DOC)
