# Supplementary figures and images for: Impact on Disease Development, Genomic Location and Biological Function of Copy Number Alterations in Non-Small Cell Lung Cancer
Source: PLoS One. 2011 Aug 2;6(8):e22961. doi: 10.1371/journal.pone.0022961 (PMC3149069; doi:10.1371/journal.pone.0022961)

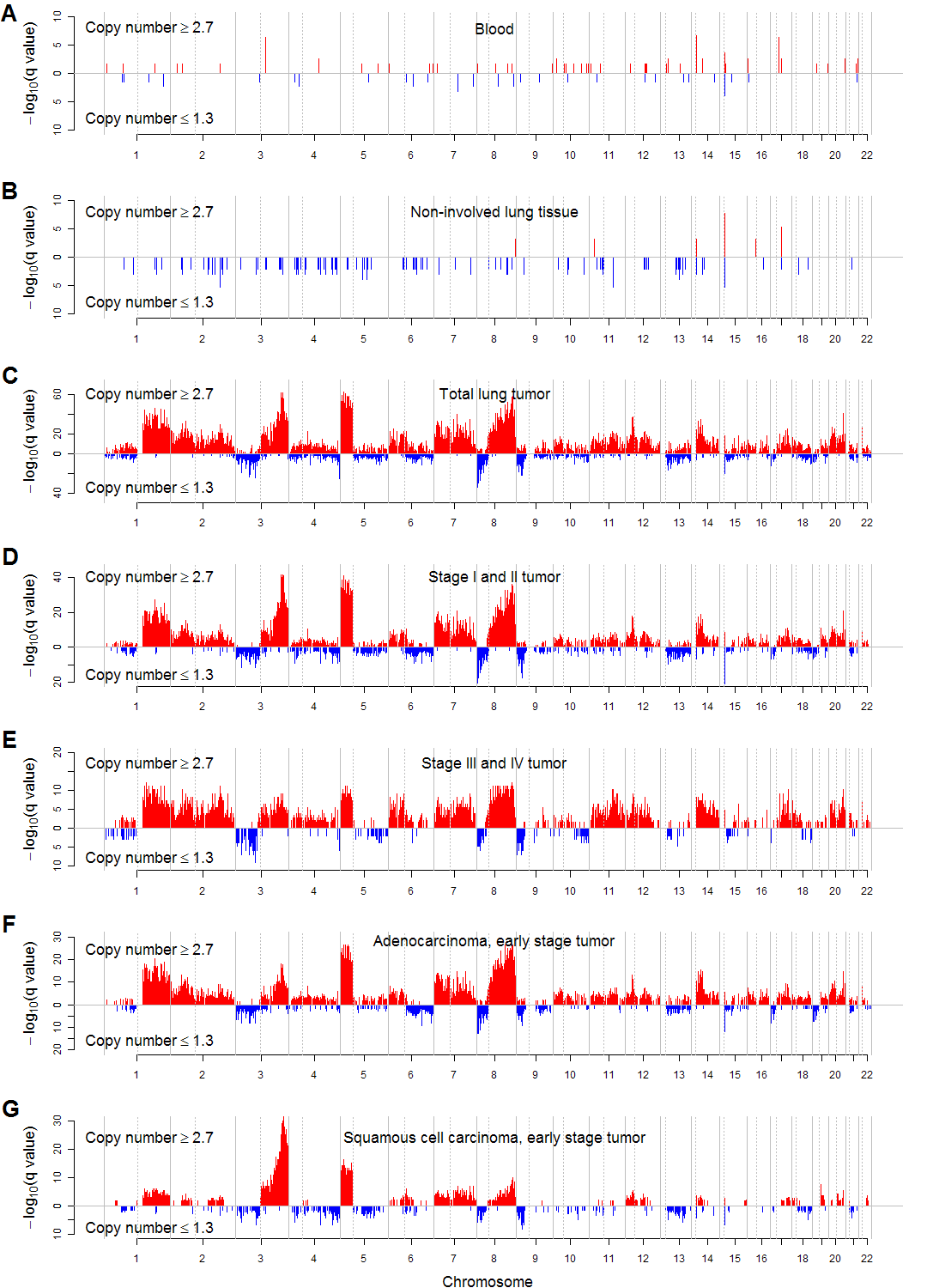

Supplement: Figure S1 — Significance of genome-wide copy number alterations in blood, non-involved lung tissue and tumor of NSCLC patients. The x axis represents genomic locations, which were ordered by the somatic chromosomes. The y axis represents −log10(q values) of NSCLC patients having copy number ≥2.7 (red) and ≤1.3 (blue) in blood (A), non-involved lung tissue (B), total tumor (C), early stage tumor (D) and late stage tumor (E), early stage tumor of adenocarcinoma (F) and early stage tumor of squamous cell carcinoma (G). (TIFF) [file pone.0022961.s001.tif]

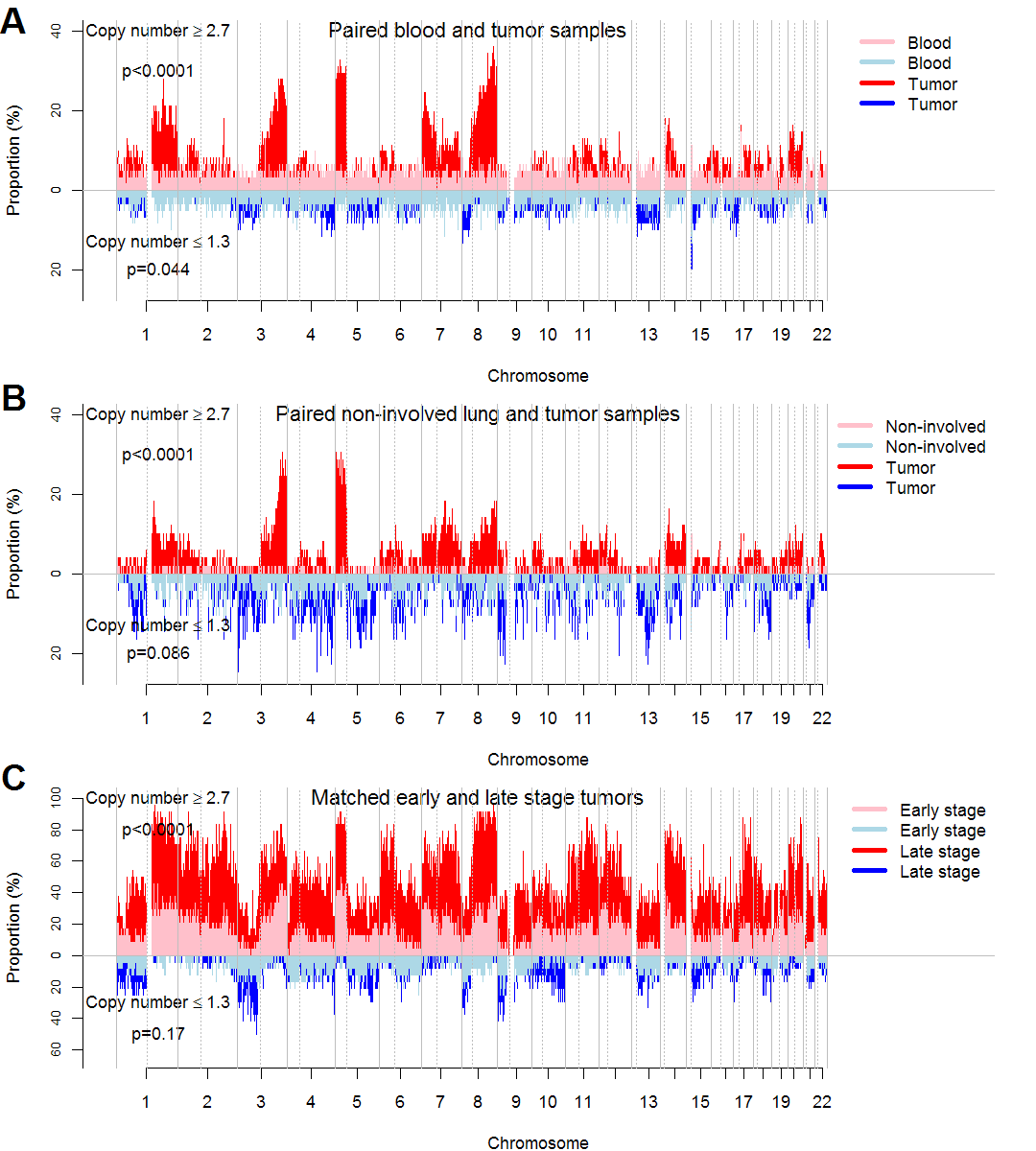

Supplement: Figure S2 — The comparisons of copy number alterations in paired or matched samples. The proportion of copy numbers ≥2.7 (red and pink) and ≤1.3 (blue and light blue) in paired blood and tumor samples (A), paired non-involved lung and tumor samples (B), and matched early stage and late stage tumors (C). The p values indicate the statistical significance from the global CNAs test between two groups. (TIFF) [file pone.0022961.s002.tif]

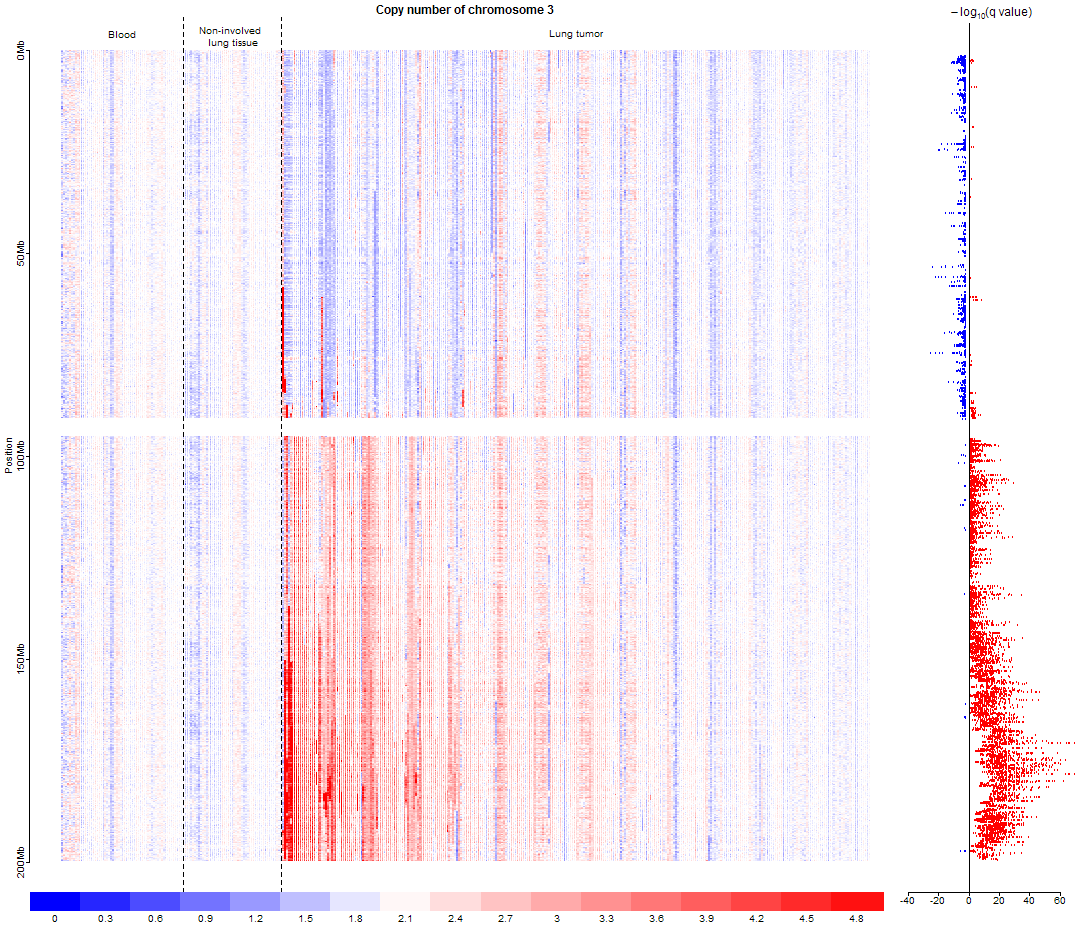

Supplement: Figure S3 — Copy numbers in chromosome 3. The main panel represents the copy numbers of blood, non-involved lung tissue and tumor as indicated. The bottom panel illustrates copy numbers with their corresponding colors (red color indicates copy number gains and blue color indicates copy number losses). The right panel shows the corresponding −log10(q values) for each locus on the chromosome; the red and blue colors indicate the −log10(q values) for copy number gains and losses, respectively. (TIFF) [file pone.0022961.s003.tif]

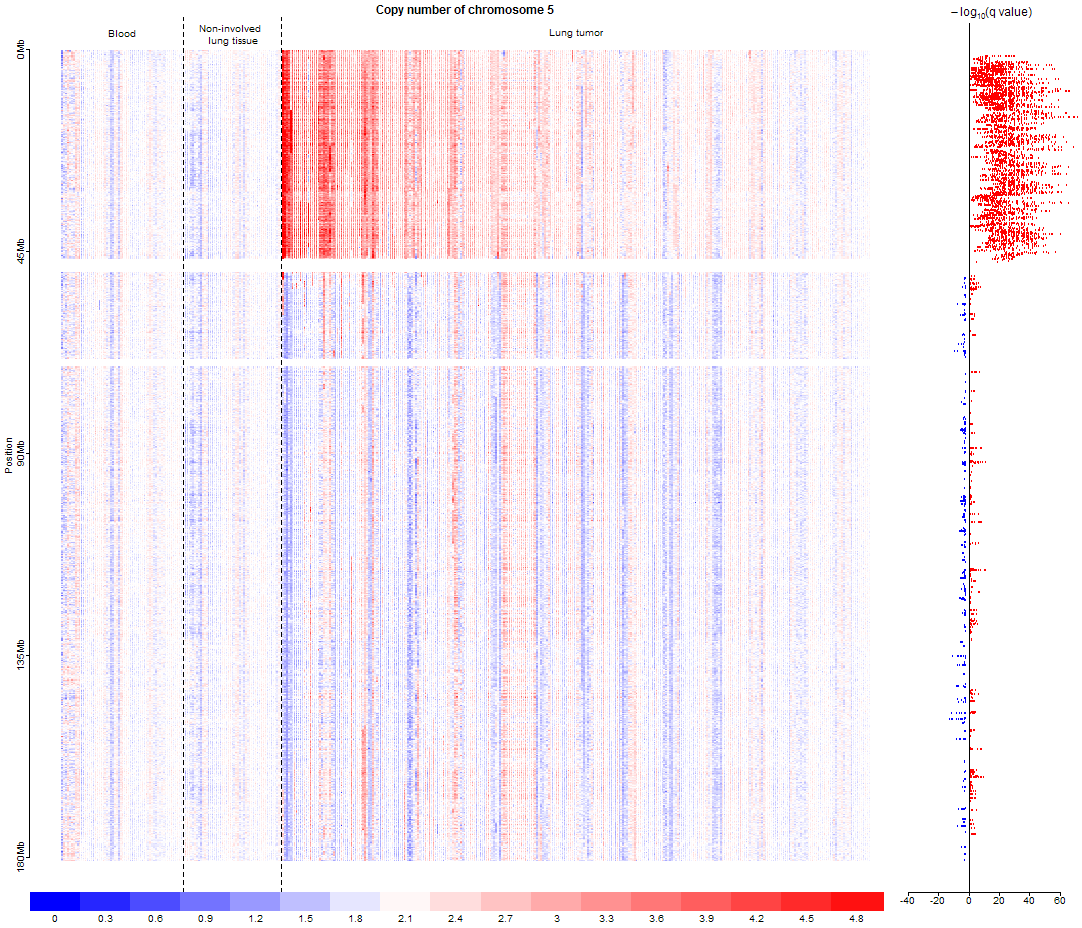

Supplement: Figure S4 — Copy numbers in chromosome 5. The main panel represents the copy numbers of blood, non-involved lung tissue and tumor as indicated. The bottom panel illustrates copy numbers with their corresponding colors (red color indicates copy number gains and blue color indicates copy number losses). The right panel shows the corresponding −log10(q values) for each locus on the chromosome; the red and blue colors indicate the −log10(q values) for copy number gains and losses, respectively. (TIFF) [file pone.0022961.s004.tif]

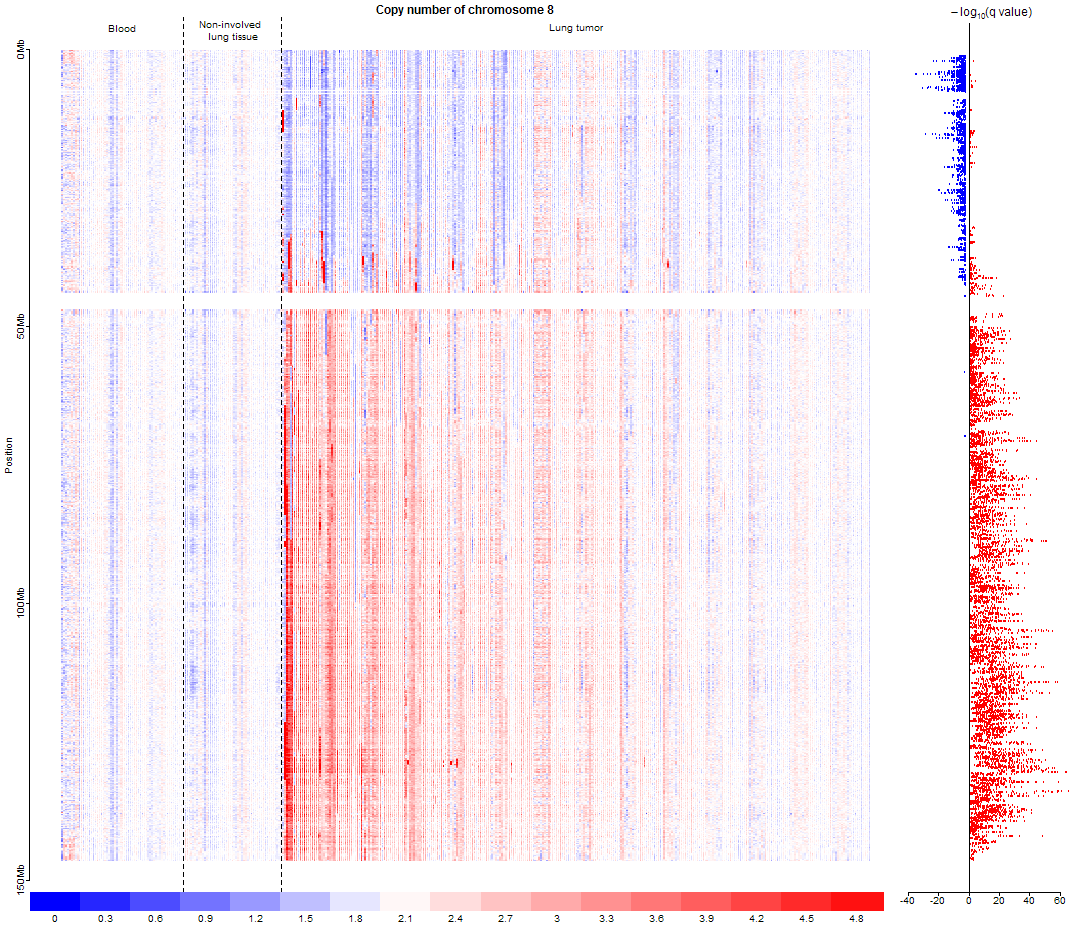

Supplement: Figure S5 — Copy numbers in chromosome 8. The main panel represents the copy numbers of blood, non-involved lung tissue and tumor as indicated. The bottom panel illustrates copy numbers with their corresponding colors (red color indicates copy number gains and blue color indicates copy number losses). The right panel shows the corresponding −log10(q values) for each locus on the chromosome; the red and blue colors indicate the −log10(q values) for copy number gains and losses, respectively. (TIFF) [file pone.0022961.s005.tif]

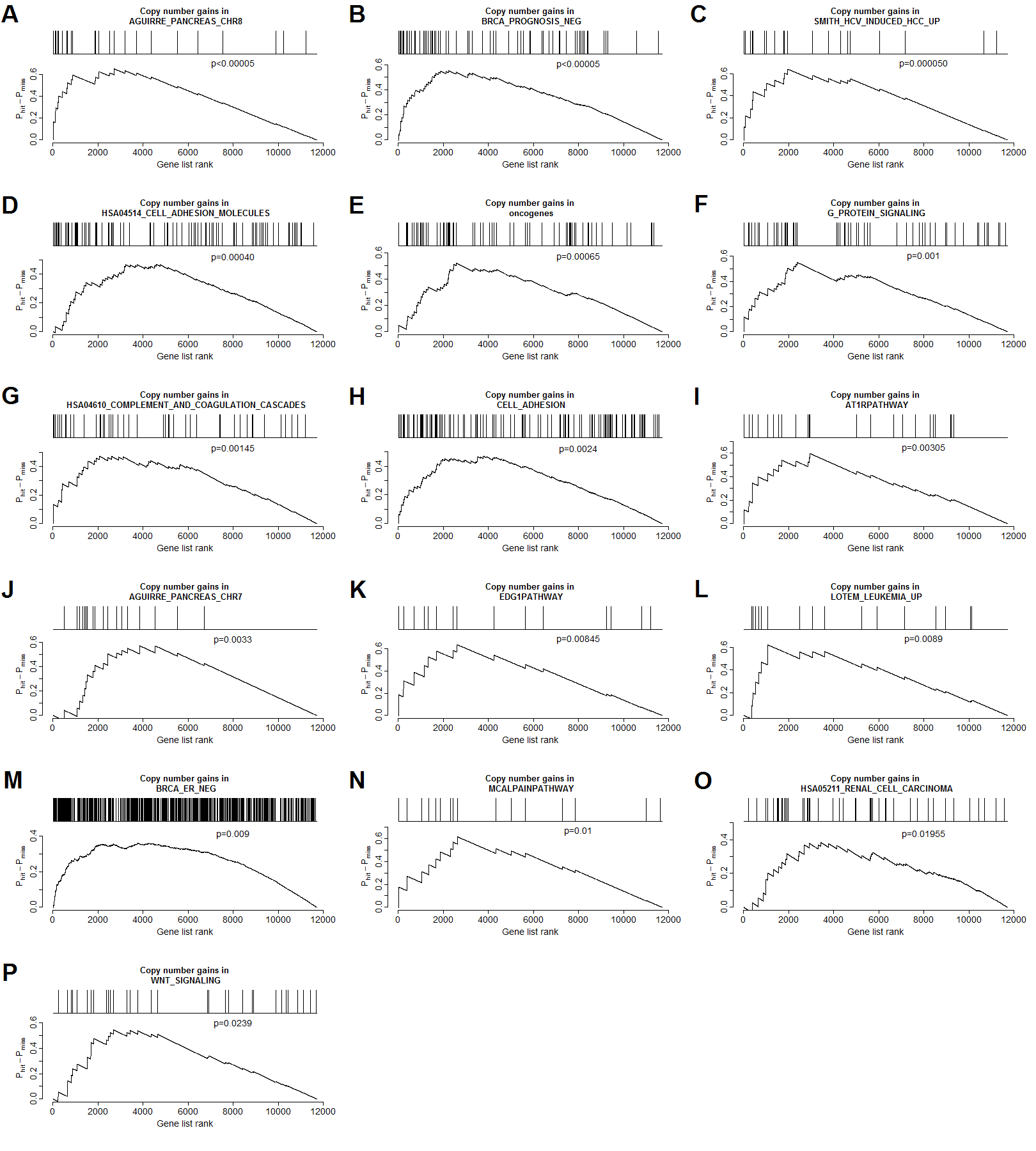

Supplement: Figure S6 — Gene set enrichment plot for the 16 selective gene sets with copy number gains (corresponding to Table 3). For each panel, the top indicates the location of genes from the gene set within the list sorted by the proportion of patients with copy number gains (left to right: higher proportion to lower); the bottom is the plot of running enrichment scores. (TIFF) [file pone.0022961.s006.tif]

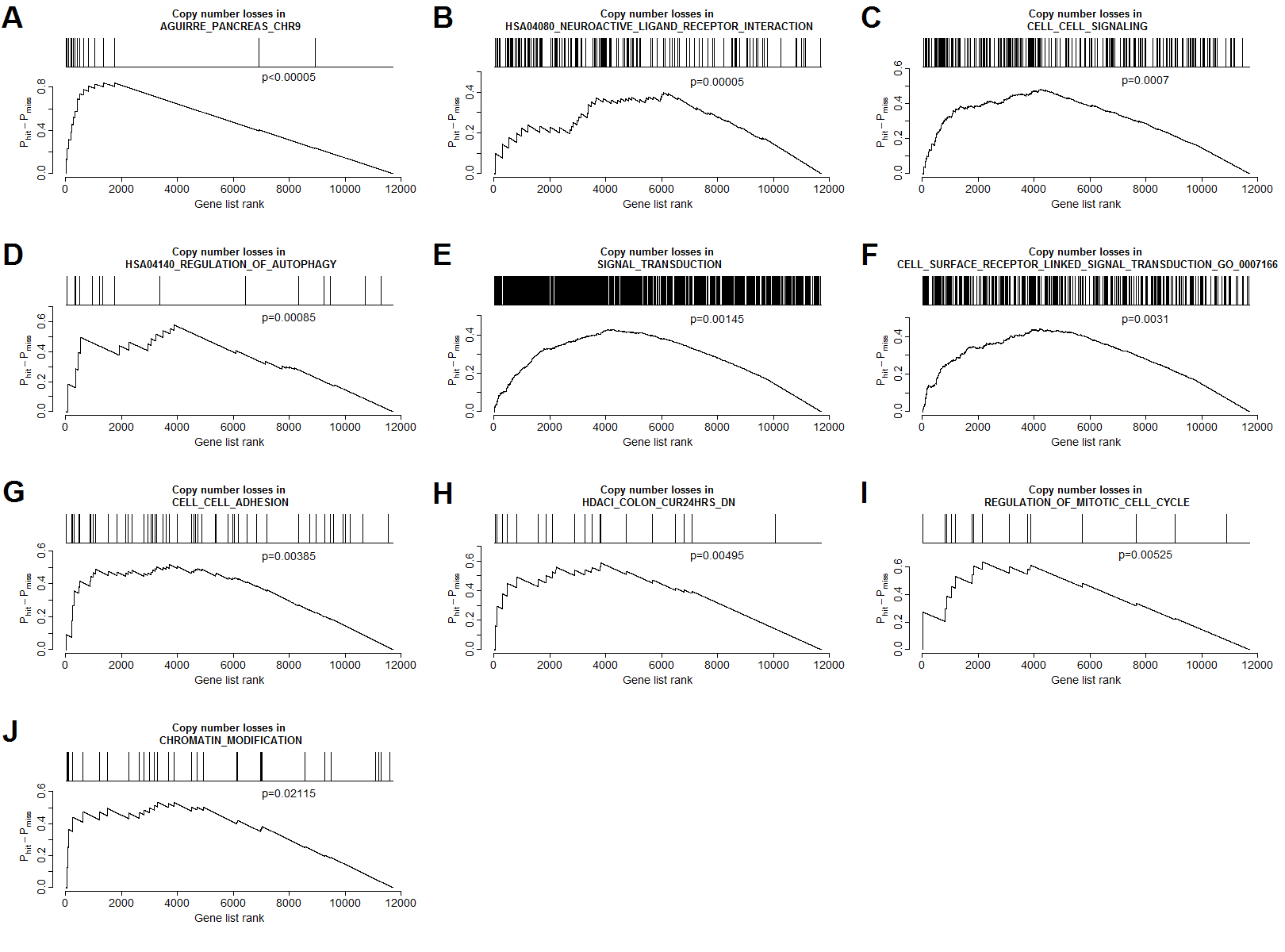

Supplement: Figure S7 — Gene set enrichment plot for the selective 10 gene sets with copy number losses (corresponding to Table 4). For each panel, the top indicates the location of genes from the gene set within the list sorted by the proportion of patients with copy number losses; the bottom is the plot of running enrichment scores. (TIFF) [file pone.0022961.s007.tif]
