# Supplementary material for: Impact on Disease Development, Genomic Location and Biological Function of Copy Number Alterations in Non-Small Cell Lung Cancer
Source: PLoS One. 2011 Aug 2;6(8):e22961. doi: 10.1371/journal.pone.0022961 (PMC3149069; doi:10.1371/journal.pone.0022961)
Supplement: Table S1 — Characteristics of the discovery and validation tumor samples*. (DOC) [file pone.0022961.s008.doc]

Table S1. Characteristics of the discovery and validation tumor samples^*^.

|  |  | Discovery set | Validation set | P value^†^ |
| --- | --- | --- | --- | --- |
| Sample size | | 151 | 150 |  |
| Clinical stage | |  |  |  |
|  | I (%) | 100 (72.5) | 99 (74.4) | 0.81 |
|  | II (%) | 23 (16.7) | 24 (18.0) |  |
|  | III and IV (%) | 15 (10.9) | 10 (7.5) |  |
| Gender | |  |  |  |
|  | Female (%) | 57 (41.0) | 50 (37.3) | 0.62 |
|  | Male (%) | 82 (59.0) | 84 (62.7) |  |
| Cell type | |  |  |  |
|  | Adenocarcinoma (%) | 103 (68.2) | 105 (70.0) | 0.83 |
|  | Squamous cell carcinoma (%) | 48 (31.8) | 45 (30.0) |  |
| Adjuvant therapy | |  |  |  |
|  | None (%) | 105 (86.8) | 108 (90.8) | 0.34 |
|  | Radiotherapy only (%) | 8 (6.6) | 7 (5.9) |  |
|  | Chemotherapy only (%) | 0 (0.0) | 1 (0.8) |  |
|  | Both (%) | 8 (6.6) | 3 (2.5) |  |
| Age | |  |  |  |
|  | Mean ± standard deviation | 67.0±8.9 | 67.5±8.1 | 0.66 |
| Smoking pack-years | |  |  |  |
|  | Median ± interquartile range | 35.3±38.0 | 35.6±40.6 | 0.70 |

^*^There are 30, 28, 61, 28 and 37 patients missing information on clinical stage, gender, adjuvant therapy, age and smoking pack-years, respectively.

^†^P values were calculated with *X^2^* test for percentage of different clinical stages (2 degree of freedom, df), male (1 df), adenocarcinoma (1 df) and different adjuvant therapy categories (3 df); with t test for age; and with Wilcoxon test for cigarette smoking pack-years.
