# Supplementary material for: Impact on Disease Development, Genomic Location and Biological Function of Copy Number Alterations in Non-Small Cell Lung Cancer
Source: PLoS One. 2011 Aug 2;6(8):e22961. doi: 10.1371/journal.pone.0022961 (PMC3149069; doi:10.1371/journal.pone.0022961)
Supplement: Table S2 — Forty-five genes with >35% patients having copy number gains. (DOC) [file pone.0022961.s009.doc]

Table S2. Forty-five genes with >35% patients having copy number gains.

|  | Symbol | Full name | Position | % of patients with copy number gains | P value^*^ |
| --- | --- | --- | --- | --- | --- |
| 1 | *DNAH5* | dynein, axonemal, heavy chain 5 | 5p15.2 | 44.85 | 5.73×10^-69^ |
| 2 | *MDS1* | myelodysplasia syndrome 1 | 3q26 | 44.19 | 5.50×10^-67^ |
| 3 | *PHF20L1* | PHD finger protein 20-like 1 | 8q24.22 | 42.86 | 4.30×10^-63^ |
| 4 | *KCNMB2* | potassium large conductance calcium-activated channel, subfamily M, beta member 2 | 3q26.2-q27.1 | 41.86 | 3.11×10^-60^ |
| 5 | *ADCY2* | adenylate cyclase 2 (brain) | 5p15.3 | 41.20 | 2.34×10^-58^ |
| 6 | *NLGN1* | neuroligin 1 | 3q26.31 | 40.86 | 1.99×10^-57^ |
| 7 | *ADAMTS16* | ADAM metallopeptidase with thrombospondin type 1 motif, 16 | 5p15 | 40.86 | 1.99×10^-57^ |
| 8 | *CTNND2* | catenin (cadherin-associated protein), delta 2 | 5p15.2 | 40.86 | 1.99×10^-57^ |
| 9 | *ADCY8* | adenylate cyclase 8 (brain) | 8q24 | 40.86 | 1.99×10^-57^ |
| 10 | *CSMD3* | CUB and Sushi multiple domains 3 | 8q23.3 | 40.53 | 1.67×10^-56^ |
| 11 | *CDH18* | cadherin 18, type 2 | 5p15.2-p15.1 | 39.87 | 1.12×10^-54^ |
| 12 | *RIMS2* | regulating synaptic membrane exocytosis 2 | 8q22.3 | 39.53 | 9.05×10^-54^ |
| 13 | *EVI1* | ecotropic viral integration site 1 | 3q24-q28 | 39.20 | 7.18×10^-53^ |
| 14 | *NAALADL2* | N-acetylated alpha-linked acidic dipeptidase-like 2 | 3q26.31 | 39.20 | 7.18×10^-53^ |
| 15 | *AGXT2* | alanine-glyoxylate aminotransferase 2 | 5p13 | 38.87 | 5.62×10^-52^ |
| 16 | *ECT2* | epithelial cell transforming sequence 2 oncogene | 3q26.1-q26.2 | 38.54 | 4.33×10^-51^ |
| 17 | *SEMA5A* | sema domain, seven thrombospondin repeats, transmembrane domain and short cytoplasmic domain, (semaphorin) 5A | 5p15.2 | 38.21 | 3.30×10^-50^ |
| 18 | *TRIO* | triple functional domain | 5p15.2 | 38.21 | 3.30×10^-50^ |
| 19 | *HEATR7B2* | HEAT repeat family member 7B2 | 5p13.1 | 38.21 | 3.30×10^-50^ |
| 20 | *CNBD1* | cyclic nucleotide binding domain containing 1 | 8q21.3 | 38.21 | 3.30×10^-50^ |
| 21 | *SPATA16* | spermatogenesis associated 16 | 3q26.31 | 37.87 | 2.48×10^-49^ |
| 22 | *GHR* | growth hormone receptor | 5p13-p12 | 37.87 | 2.48×10^-49^ |
| 23 | *DNAH14* | dynein, axonemal, heavy chain 14 | 1q42.12 | 37.54 | 1.83×10^-48^ |
| 24 | *OSMR* | oncostatin M receptor | 5p13.1 | 37.54 | 1.83×10^-48^ |
| 25 | *C6* | complement component 6 | 5p13 | 37.54 | 1.83×10^-48^ |
| 26 | *CMBL* | carboxymethylenebutenolidase homolog | 5p15.2 | 37.21 | 1.34×10^-47^ |
| 27 | *FYB* | FYN binding protein | 5p13.1 | 37.21 | 1.34×10^-47^ |
| 28 | *NSMCE2* | non-SMC element 2, MMS21 homolog | 8q24.13 | 37.21 | 1.34×10^-47^ |
| 29 | *PTK2* | PTK2 protein tyrosine kinase 2 | 8q24-qter | 37.21 | 1.34×10^-47^ |
| 30 | *SLC7A14* | solute carrier family 7, member 14 | 3q26.2 | 36.88 | 9.62×10^-47^ |
| 31 | *KCNAB1* | potassium voltage-gated channel, shaker-related subfamily, beta member 1 | 3q26.1 | 36.54 | 6.82×10^-46^ |
| 32 | *CDH10* | cadherin 10, type 2 | 5p14-p13 | 36.54 | 6.82×10^-46^ |
| 33 | *RSRC1* | arginine/serine-rich coiled-coil 1 | 3q25.32 | 36.21 | 4.77×10^-45^ |
| 34 | *ADAMTS12* | ADAM metallopeptidase with thrombospondin type 1 motif, 12 | 5q35 | 36.21 | 4.77×10^-45^ |
| 35 | *WDSOF1* | WD repeats and SOF1 domain containing | 8q22.3 | 36.21 | 4.77×10^-45^ |
| 36 | *DNM3* | dynamin 3 | 1q24.3 | 35.88 | 3.29×10^-44^ |
| 37 | *SLC1A3* | solute carrier family 1, member 3 | 5p13 | 35.88 | 3.29×10^-44^ |
| 38 | *BCAS1* | breast carcinoma amplified sequence 1 | 20q13.2 | 35.88 | 3.29×10^-44^ |
| 39 | *XPR1* | xenotropic and polytropic retrovirus receptor | 1q25.1 | 35.55 | 2.24×10^-43^ |
| 40 | *FAM134B* | family with sequence similarity 134, member B | 5p15.1 | 35.55 | 2.24×10^-43^ |
| 41 | *PRLR* | prolactin receptor | 5p13.2 | 35.55 | 2.24×10^-43^ |
| 42 | *OXR1* | oxidation resistance 1 | 8q23 | 35.55 | 2.24×10^-43^ |
| 43 | *C7* | complement component 7 | 5p13 | 35.22 | 1.50×10^-42^ |
| 44 | *CCDC152* | coiled-coil domain containing 152 | 5p12 | 35.22 | 1.50×10^-42^ |
| 45 | *TG* | Thyroglobulin | 8q24 | 35.22 | 1.50×10^-42^ |

^*^ *P* values were calculated to test the significance of observing the percentage (%) of patients with copy number gains (copy number ≥ 2.7) on the oncogenes given that the null probability of copy number gains is 0.076, which is empirically estimated from the data.
