# Supplementary material for: Impact on Disease Development, Genomic Location and Biological Function of Copy Number Alterations in Non-Small Cell Lung Cancer
Source: PLoS One. 2011 Aug 2;6(8):e22961. doi: 10.1371/journal.pone.0022961 (PMC3149069; doi:10.1371/journal.pone.0022961)
Supplement: Table S3 — Eighty-nine pathways and gene sets with enrichment of genes with copy number gains (with p<0.05 in discovery set, validation set, early stage and late stage tumors). (DOC) [file pone.0022961.s010.doc]

Table S3. Eighty-nine pathways and gene sets with enrichment of genes with copy number gains (with p <0.05 in discovery set, validation set, early stage and late stage tumors).

|  | MSigDB ID of gene set: brief description | P value in blood (n=63) | P value in non-involved lung (n=50) | P value in discovery set (n=151) | P value in validation set (n=150) | P value in early stage tumors (n=246) | P value in late stage tumors (n=25) | P value in all tumors (n=301) |
| --- | --- | --- | --- | --- | --- | --- | --- | --- |
| 1 | CREB_BRAIN_8WKS_UP: Up-regulated in the nucleus accumbens of mice after 8 weeks of induction of transgenic CREB | 0.13 | <0.00005 | <0.00005 | 0.000050 | <0.00005 | 0.0036 | <0.00005 |
| 2 | CALCIUM_REGULATION_IN_CARDIAC_CELLS: Genes in calcium regulation in cardiac cells | 0.94 | 0.33 | 0.00005 | <0.00005 | 0.000050 | 0.00010 | <0.00005 |
| 3 | AGUIRRE_PANCREAS_CHR8: Genes on chromosome 8 with copy-number-driven expression in pancreatic adenocarcinoma. | 0.46 | <0.00005 | <0.00005 | <0.00005 | <0.00005 | 0.000050 | <0.00005 |
| 4 | DIAB_NEPH_DN: Downregulated in the glomeruli of cadaver kidneys from patients with diabetic nephropathy, compared to normal controls | 0.97 | 0.85 | 0.0010 | <0.00005 | 0.00080 | <0.00005 | <0.00005 |
| 5 | UVC_TTD_4HR_DN: Down-regulated at 4 hours following treatment of XPB/TTD fibroblasts with 3 J/m^2^ UVC | 0.93 | <0.00005 | <0.00005 | <0.00005 | 0.00035 | <0.00005 | <0.00005 |
| 6 | UVC_XPCS_4HR_DN: Down-regulated at 4 hours following treatment of XPB/CS fibroblasts with 3 J/m^2^ UVC | 0.83 | <0.00005 | 0.00030 | <0.00005 | 0.00035 | <0.00005 | <0.00005 |
| 7 | TAKEDA_NUP8_HOXA9_6H_UP: Effect of NUP98-HOXA9 on gene transcription at 6 h after transfection UP | 0.14 | 0.36 | <0.00005 | <0.00005 | 0.00025 | <0.00005 | <0.00005 |
| 8 | UVC_XPCS_ALL_DN: Down-regulated at any timepoint following treatment of XPB/CS fibroblasts with 3 J/m^2^ UVC | 0.99 | 0.99 | <0.00005 | <0.00005 | 0.00004 | <0.00005 | <0.00005 |
| 9 | UVC_TTD_8HR_DN: Down-regulated at 8 hours following treatment of XPB/TTD fibroblasts with 3 J/m^2^ UVC | 0.96 | 0.72 | <0.00005 | <0.00005 | 0.00004 | <0.00005 | <0.00005 |
| 10 | BRCA_PROGNOSIS_NEG: Genes whose expression is consistently negatively correlated with breast cancer outcomes - higher expression is associated with metastasis and poor prognosis | 0.64 | <0.00005 | <0.00005 | <0.00005 | 0.00004 | <0.00005 | <0.00005 |
| 11 | POD1_KO_DN: Down-regulated in glomeruli isolated from Pod1 knockout mice, versus wild-type controls | 0.54 | 0.98 | <0.00005 | <0.00005 | 0.00004 | <0.00005 | <0.00005 |
| 12 | UVC_TTD_ALL_DN: Down-regulated at any timepoint following treatment of XPB/TTD fibroblasts with 3 J/m^2^ UVC | 0.99 | 0.97 | <0.00005 | <0.00005 | 0.00004 | <0.00005 | <0.00005 |
| 13 | UVC_XPCS_8HR_DN: Down-regulated at 8 hours following treatment of XPB/CS fibroblasts with 3 J/m^2^ UVC | 0.99 | 0.98 | <0.00005 | <0.00005 | 0.00004 | <0.00005 | <0.00005 |
| 14 | SMITH_HCV_INDUCED_HCC_UP: Genes highly expressed in hepatitis C-related hepatocellular carcinoma | 0.57 | <0.00005 | 0.00025 | 0.00005 | 0.00004 | 0.00085 | 0.000050 |
| 15 | BOQUEST_CD31PLUS_VS_CD31MINUS_UP: Genes overexpressed 3-fold or more in freshly isolated CD31+ versus freshly isolated CD31- cells | 0.64 | 0.79 | 0.00010 | 0.0001 | 0.00010 | <0.00005 | 0.00010 |
| 16 | HADDAD_HSC_CD7_DN: Genes upregulated in human hematopoietic stem cells of the line CD45RA(int) CD7- versus CD45RA(hi) CD7+. | 0.83 | <0.00005 | 0.00015 | 0.00035 | 0.00050 | 0.0026 | 0.00015 |
| 17 | HADDAD_CD45CD7_PLUS_VS_MINUS_DN: Genes enriched in CD45RAintCD7- vs CD45RAhiCD7hi HPCs | 0.83 | <0.00005 | 0.00015 | 0.00035 | 0.00050 | 0.0026 | 0.00015 |
| 18 | GH_AUTOCRINE_DN: Down-regulated following stable autocrine expression of human growth hormone in mammary carcinoma cells (MCF-7) | 0.72 | 0.68 | 0.00015 | 0.00025 | 0.000040 | 0.00035 | 0.00020 |
| 19 | ALZHEIMERS_DISEASE_DN: Downregulated in correlation with overt Alzheimer's Disease, in the CA1 region of the hippocampus | 0.83 | 1.0 | 0.0019 | 0.00015 | 0.0021 | 0.0014 | 0.00030 |
| 20 | HSA04514_CELL_ADHESION_MOLECULES: Genes involved in cell adhesion molecules (CAMs) | 0.029 | 0.39 | 0.00085 | 0.00040 | 0.0049 | 0.00025 | 0.00040 |
| 21 | AGED_RHESUS_UP: Upregulated in the vastus lateralis muscle of aged vs. young adult rhesus monkeys | 0.36 | 0.31 | 0.00015 | 0.00070 | 0.0031 | 0.000050 | 0.00055 |
| 22 | AGEING_KIDNEY_DN: Down-regulation is associated with increasing age in normal human kidney tissue from 74 patients | 0.010 | <0.00005 | 0.00035 | 0.0012 | 0.0004 | 0.024 | 0.00065 |
| 23 | ONCOGENE: Census of human cancer genes | 0.37 | 0.51 | 0.0011 | 0.0047 | 0.0018 | 0.015 | 0.00065 |
| 24 | HSC_HSC_FETAL: Up-regulated in mouse hematopoietic stem cells from fetal liver (HSC Shared + Fetal) | 0.63 | 0.69 | 0.00095 | 0.00075 | 0.0023 | 0.00075 | 0.00070 |
| 25 | UVC_TTD-XPCS_COMMON_DN: Down-regulated at any timepoint following treatment of both XPB/CS and XPB/TTD fibroblasts with 3 J/m^2^ UVC | 0.85 | 0.66 | 0.0030 | 0.00045 | 0.011 | 0.00005 | 0.00090 |
| 26 | G_PROTEIN_SIGNALING: Gene involved in G protein signaling | 0.41 | <0.00005 | 0.0016 | 0.0021 | 0.0030 | 0.010 | 0.0010 |
| 27 | BRENTANI_CELL_ADHESION: Cancer related genes involved in cell adhesion and metalloproteinases | 0.034 | 0.57 | 0.0027 | 0.0016 | 0.0024 | 0.0050 | 0.0011 |
| 28 | POTASSIUM_ION_TRANSPORT: Genes annotated by the GO term GO:0006813. The directed movement of potassium ions (K^+^) into, out of, within or between cells. | 0.59 | <0.00005 | 0.0012 | 0.0033 | 0.0009 | 0.048 | 0.0012 |
| 29 | BAF57_BT549_UP: Up-regulated following stable re-expression of BAF57 in Bt549 breast cancer cells that lack functional BAF57 | 0.66 | 0.35 | 0.0022 | 0.0021 | 0.017 | 0.0019 | 0.0013 |
| 30 | MONOVALENT_INORGANIC_CATION_TRANSPORT: Genes annotated by the GO term GO:0015672. The directed movement of inorganic cations with a valency of one into, out of, within or between cells. Inorganic cations are atoms or small molecules with a positive charge which do not contain carbon in covalent linkage. | 0.60 | <0.00005 | 0.00085 | 0.0056 | 0.0011 | 0.012 | 0.0014 |
| 31 | HSA04610_COMPLEMENT_AND_COAGULATION  _CASCADES: Genes involved in complement and coagulation cascades | 0.14 | <0.00005 | 0.0012 | 0.0028 | 0.00065 | 0.0036 | 0.0015 |
| 32 | BOQUEST_CD31PLUS_VS_CD31MINUS_DN: Genes overexpressed 3-fold or more in freshly isolated CD31- versus freshly isolated CD31+ cells | 0.69 | <0.00005 | 0.0045 | 0.0016 | 0.0073 | 0.0023 | 0.0018 |
| 33 | HSC_HSC_SHARED: Up-regulated in mouse hematopoietic stem cells from both adult bone marrow and fetal liver (Cluster ii, HSC Shared) | 0.57 | 0.65 | 0.0021 | 0.0021 | 0.0064 | 0.0028 | 0.0020 |
| 34 | GH_AUTOCRINE_UP: Up-regulated following stable autocrine expression of human growth hormone in mammary carcinoma cells (MCF-7) | 0.33 | 0.50 | 0.0017 | 0.0027 | 0.018 | 0.0021 | 0.0021 |
| 35 | SMOOTH_MUSCLE_CONTRACTION: Genes involved in smooth muscle contraction | 0.80 | 0.32 | 0.0051 | 0.0017 | 0.0054 | 0.021 | 0.0022 |
| 36 | ROSS_FAB_M7: Genes upregulated in AML samples of the FAB class M7 | 0.52 | <0.00005 | 0.0011 | 0.0065 | 0.0049 | 0.00060 | 0.0023 |
| 37 | CELL_ADHESION: The attachment of a cell, either to another cell or to the extracellular matrix, via cell adhesion molecules. | 0.0005 | 0.76 | 0.0026 | 0.0041 | 0.012 | 0.0012 | 0.0024 |
| 38 | ET743PT650_COLONCA_DN: Downregulated by both Et-743 and Pt-650 in HCT116 cells | 0.61 | <0.00005 | 0.0050 | 0.00090 | 0.028 | 0.000050 | 0.0030 |
| 39 | AT1RPATHWAY: Binding of angiotensin II to AT1-R activates Ca2+ signaling and the JNK pathway. | 0.0005 | <0.00005 | 0.0041 | 0.0038 | 0.0068 | 0.016 | 0.0031 |
| 40 | VANTVEER_BREAST_OUTCOME_GOOD_VS_POOR  _DN: Poor prognosis marker genes in Breast Cancer (part of NKI-70) from Van't Veer et al 2002 | 0.59 | 0.32 | 0.0038 | 0.0030 | 0.0002 | 0.0057 | 0.0033 |
| 41 | AGUIRRE_PANCREAS_CHR7: Genes on chromosome 7 with copy-number-driven expression in pancreatic adenocarcinoma. | 0.93 | <0.00005 | 0.018 | 0.00025 | 0.0026 | 0.0019 | 0.0033 |
| 42 | MULTICELLULAR_ORGANISMAL_DEVELOPMENT: Genes annotated by the GO term GO:0007275. The biological process whose specific outcome is the progression of an organism over time from an initial condition (e.g. a zygote or a young adult) to a later condition (e.g. a multicellular animal or an aged adult). | 0.095 | 0.74 | 0.016 | 0.0025 | 0.043 | 0.014 | 0.0036 |
| 43 | HSC_LTHSC_ADULT: Up-regulated in mouse long-term functional hematopoietic stem cells from adult bone marrow (LT-HSC Shared + Adult) | 0.49 | 0.50 | 0.0061 | 0.0019 | 0.011 | 0.037 | 0.0036 |
| 44 | HSC_HSC_ADULT: Up-regulated in mouse hematopoietic stem cells from adult bone marrow (HSC Shared + Adult) | 0.59 | 0.80 | 0.010 | 0.0033 | 0.012 | 0.014 | 0.0037 |
| 45 | HSC_LTHSC_SHARED: Up-regulated in mouse long-term functional hematopoietic stem cells from both adult bone marrow and fetal liver (Cluster i, LT-HSC Shared) | 0.75 | 0.73 | 0.0062 | 0.0040 | 0.016 | 0.045 | 0.0037 |
| 46 | HSC_LTHSC_FETAL: Up-regulated in mouse long-term functional hematopoietic stem cells from fetal liver (LT-HSC Shared) | 0.75 | 0.73 | 0.0062 | 0.0040 | 0.016 | 0.045 | 0.0037 |
| 47 | VERHAAK_AML_NPM1_MUT_VS_WT_UP: Genes that are upregulated in AML NPM1 mutant versus AML NPM1 wild type | 0.93 | <0.00005 | 0.0043 | 0.012 | 0.008 | 0.0079 | 0.0042 |
| 48 | DFOSB_BRAIN_2WKS_UP: Up-regulated in the nucleus accumbens of mice after 2 weeks of induction of transgenic deltaFosB | 0.083 | <0.00005 | 0.0047 | 0.0039 | 0.0045 | 0.022 | 0.0059 |
| 49 | UVB_NHEK3_C8: Regulated by UV-B light in normal human epidermal keratinocytes, cluster 8 | 0.86 | 0.36 | 0.011 | 0.0027 | 0.0019 | 0.0075 | 0.0063 |
| 50 | REGULATION_OF_NEUROTRANSMITTER_LEVELS: Genes annotated by the GO term GO:0001505. Any process that modulates levels of neurotransmitter. | 0.15 | <0.00005 | 0.0088 | 0.0060 | 0.0095 | 0.019 | 0.0064 |
| 51 | ALCALAY_AML_NPMC_UP: Increased expression in NPMc+ leukemias | 0.91 | 0.49 | 0.030 | 0.0060 | 0.013 | 0.015 | 0.0066 |
| 52 | UV_UNIQUE_FIBRO_DN: Down-regulated at any timepoint by treatment of human fibroblasts with UV light, but not bygamma radiation or 4-NQO | 0.012 | <0.00005 | 0.0065 | 0.0059 | 0.010 | 0.010 | 0.0069 |
| 53 | UV-CMV_UNIQUE_HCMV_6HRS_UP: Up-regulated in fibroblasts at 6 hours following infection with UV-inactivated CMV, but not untreated CMV | 0.48 | <0.00005 | 0.019 | 0.013 | 0.012 | 0.019 | 0.0073 |
| 54 | UVB_NHEK1_DN: Downregulated by UV-B light in normal human epidermal keratinocytes | 0.77 | 0.86 | 0.019 | 0.0025 | 0.024 | 0.0020 | 0.0077 |
| 55 | EDG1PATHWAY: The lipid S1P is an EDG1 ligand promoting chemotaxis via Rac1 and cell survival and proliferation via ERK activation. | 0.031 | <0.00005 | 0.015 | 0.0089 | 0.010 | 0.022 | 0.0085 |
| 56 | CMV_HCMV_TIMECOURSE_48HRS_UP: Up-regulated in fibroblasts following infection with human cytomegalovirus (at least 3-fold, with Affymetrix change call, in at least two consectutive timepoints), with maximum change at 48 hours | 0.88 | <0.00005 | 0.0026 | 0.014 | 0.022 | 0.014 | 0.0088 |
| 57 | LOTEM_LEUKEMIA_UP: Genes upregulated in myeloid leukemia and normally expressed in other bodily tissues. | 0.62 | <0.00005 | 0.0048 | 0.033 | 0.0077 | 0.041 | 0.0089 |
| 58 | BRCA_ER_NEG: Genes whose expression is consistently negatively correlated with estrogen receptor status in breast cancer - higher expression is associated with ER-negative tumors | 0.85 | 0.94 | 0.0069 | 0.0099 | 0.0037 | 0.012 | 0.009 |
| 59 | NOUZOVA_CPG_METHLTD: Aberrantly methylated CpG islands in NB4 (ranks 1 and 2 only) | 0.066 | <0.00005 | 0.015 | 0.0022 | 0.013 | 0.050 | 0.0094 |
| 60 | YAGI_AML_PROG_FAB: FAB type-specific probe sets | 0.74 | 0.79 | 0.0036 | 0.017 | 0.030 | 0.00015 | 0.010 |
| 61 | MCALPAINPATHWAY: In integrin-mediated cell migration, calpains digest links between the actin cytoskeleton and focal adhesion proteins. | 0.80 | <0.00005 | 0.019 | 0.0080 | 0.0078 | 0.047 | 0.010 |
| 62 | IRITANI_ADPROX_DN: BEC-specific suppressed by AdProx-1 | 0.77 | <0.00005 | 0.0026 | 0.028 | 0.020 | 0.0040 | 0.010 |
| 63 | HDACI_COLON_BUT_DN: Downregulated by butyrate at any timepoint up to 48 hrs in SW260 colon carcinoma cells | 0.94 | 0.56 | 0.0087 | 0.011 | 0.0035 | 0.011 | 0.013 |
| 64 | ION_TRANSPORT: Genes annotated by the GO term GO:0006811. The directed movement of charged atoms or small charged molecules into, out of, within or between cells. | 0.23 | 0.49 | 0.016 | 0.014 | 0.029 | 0.034 | 0.013 |
| 65 | HEMOPOIESIS: Genes annotated by the GO term GO:0030097. The process whose specific outcome is the progression of the myeloid and lymphoid derived organ/tissue systems of the blood and other parts of the body over time, from formation to the mature structure. The site of hemopoiesis is variable during development, but occurs primarily in bone marrow or kidney in many adult vertebrates. | 0.94 | <0.00005 | 0.014 | 0.011 | 0.014 | 0.018 | 0.015 |
| 66 | HEMOPOIETIC_OR_LYMPHOID_ORGAN  _DEVELOPMENT: Genes annotated by the GO term GO:0048534. The process whose specific outcome is the progression of any organ involved in hemopoiesis or lymphoid cell activation over time, from its formation to the mature structure. Such development includes differentiation of resident cell types (stromal cells) and of migratory cell types dependent on the unique microenvironment afforded by the organ for their proper differentiation. | 0.94 | <0.00005 | 0.014 | 0.011 | 0.014 | 0.018 | 0.015 |
| 67 | DETECTION_OF_STIMULUS: Genes annotated by the GO term GO:0051606. The series of events in which a stimulus is received by a cell and converted into a molecular signal. | 0.75 | <0.00005 | 0.0088 | 0.036 | 0.012 | 0.030 | 0.015 |
| 68 | VERHAAK_AML_NPM1_MUT_VS_WT_DN: Description Genes that are downregulated in AML NPM1 mutant versus AML NPM1 wild type | 0.11 | <0.00005 | 0.030 | 0.0093 | 0.044 | 0.019 | 0.016 |
| 69 | CARIES_PULP_DN: Down-regulated in pulpal tissue from extracted carious teeth (cavities), compared to tissue from extracted healthy teeth | 0.45 | <0.00005 | 0.0078 | 0.038 | 0.035 | 0.029 | 0.018 |
| 70 | PROTEIN_IMPORT_INTO_NUCLEUS: Genes annotated by the GO term GO:0006606. The directed movement of a protein from the cytoplasm to the nucleus. | 0.27 | <0.00005 | 0.017 | 0.011 | 0.031 | 0.0054 | 0.018 |
| 71 | JNK_DN: Downregulated by expression of constitutively active JNK in 3T3 cells | 0.84 | <0.00005 | 0.012 | 0.017 | 0.0094 | 0.016 | 0.018 |
| 72 | LEE_TCELLS10_UP: Transcripts showing more than 2 fold higher expression in CB4 than in AB4 | 0.82 | 0.70 | 0.015 | 0.015 | 0.049 | 0.0068 | 0.019 |
| 73 | LEE_TCELLS8_UP: Transcripts enriched in na???ve CD4 T cells (CB4, and AB4) more than 3-fold, with average signal value differences of at least 100 between thymocytes (ITTP, DP, SP4) and naive-phenotype CD4 T (CB4, and AB4) cells | 0.82 | 0.70 | 0.015 | 0.015 | 0.049 | 0.0068 | 0.019 |
| 74 | LEE_TCELLS1_UP: Transcripts enriched in more mature cells (SP4, CB4, and AB4) more than 3-fold, with average signal value differences of at least 100 between less mature (ITTP, DP) and more mature (SP4, CB4, and AB4) cells | 0.82 | 0.70 | 0.015 | 0.015 | 0.049 | 0.0068 | 0.019 |
| 75 | LINDSTEDT_DEND_UP: Genes up-regulated in dendritic cell (DC) stimulated for 8 and 48 h | 0.91 | <0.00005 | 0.021 | 0.021 | 0.017 | 0.023 | 0.019 |
| 76 | POD1_KO_UP: Up-regulated in glomeruli isolated from Pod1 knockout mice, versus wild-type controls | 0.92 | 0.27 | 0.042 | 0.0082 | 0.036 | 0.048 | 0.019 |
| 77 | CMV-UV_HCMV_6HRS_UP: Up-regulated in fibroblasts at 6 hours following infection with UV-inactivated human cytomegalovirus | 0.34 | <0.00005 | 0.033 | 0.020 | 0.031 | 0.027 | 0.019 |
| 78 | XPB_TTD-CS_UP: Up-regulated in XPB/TTD fibroblasts (expressing XPB-A355C), compared to XPB/CS fibroblasts (expressing XPB-T296C) | 0.38 | <0.00005 | 0.029 | 0.022 | 0.041 | 0.0084 | 0.019 |
| 79 | HSA05211_RENAL_CELL_CARCINOMA: Genes involved in renal cell carcinoma | 0.32 | 0.32 | 0.023 | 0.031 | 0.0082 | 0.025 | 0.020 |
| 80 | UVC_LOW_C2_DN: Down-regulated at 6-12 hours following treatment of WS1 human skin fibroblasts with UVC at a low dose (10 J/m^2^) (cluster c2) C | 0.45 | <0.00005 | 0.042 | 0.0093 | 0.047 | 0.021 | 0.020 |
| 81 | HDACI_COLON_BUT_UP: Upregulated by butyrate at any timepoint up to 48 hrs in SW260 colon carcinoma cells | 0.44 | <0.00005 | 0.025 | 0.024 | 0.020 | 0.039 | 0.021 |
| 82 | IMMUNE_SYSTEM_DEVELOPMENT: Genes annotated by the GO term GO:0002520. The process whose specific outcome is the progression of an organismal system whose objective is to provide calibrated responses by an organism to a potential internal or invasive threat, over time, from its formation to the mature structure. A system is a regularly interacting or interdependent group of organs or tissues that work together to carry out a given biological process. | 0.96 | <0.00005 | 0.021 | 0.017 | 0.022 | 0.028 | 0.022 |
| 83 | NUCLEAR_IMPORT: Genes annotated by the GO term GO:0051170. The directed movement of substances into the nucleus. | 0.17 | <0.00005 | 0.024 | 0.015 | 0.040 | 0.0079 | 0.023 |
| 84 | WNT_SIGNALING: *Wnt* signaling genes | 0.17 | 0.31 | 0.020 | 0.025 | 0.021 | 0.0077 | 0.024 |
| 85 | AGED_MOUSE_HIPPOCAMPUS_ANY_UP: Up-regulated in the hippocampus of 16-month aged mice from any of four strains (S8, S10, SR1, B6J), versus 3-month young controls | 0.25 | <0.00005 | 0.033 | 0.033 | 0.037 | 0.0039 | 0.024 |
| 86 | HSA04742_TASTE_TRANSDUCTION: Genes involved in taste transduction | 0.33 | <0.00005 | 0.024 | 0.036 | 0.017 | 0.014 | 0.024 |
| 87 | FEMALE_PREGNANCY: Genes annotated by the GO term GO:0007565. The physiological processes that allow an embryo or foetus to develop within the body of a female animal. It covers the time from fertilization of a female ovum by a male spermatozoon until birth. | 0.97 | <0.00005 | 0.035 | 0.028 | 0.025 | 0.030 | 0.025 |
| 88 | IL1_CORNEA_UP: Upregulated in corneal fibroblasts after interleukin-1 treatment (Tables 1 and 2) | 0.19 | 0.33 | 0.028 | 0.030 | 0.028 | 0.039 | 0.032 |
| 89 | HDACI_COLON_BUT30MIN_DN: Downregulated by butyrate at 30 min in SW260 colon carcinoma cells | 0.48 | 0.13 | 0.034 | 0.045 | 0.021 | 0.028 | 0.044 |
