# Supplementary material for: Impact on Disease Development, Genomic Location and Biological Function of Copy Number Alterations in Non-Small Cell Lung Cancer
Source: PLoS One. 2011 Aug 2;6(8):e22961. doi: 10.1371/journal.pone.0022961 (PMC3149069; doi:10.1371/journal.pone.0022961)
Supplement: Table S4 — Twenty-seven pathways and gene sets with enrichment of genes with copy number losses (with p<0.05 in discovery set, validation set, early stage and late stage tumors). (DOC) [file pone.0022961.s011.doc]

Table S4. Twenty-seven pathways and gene sets with enrichment of genes with copy number losses (with p <0.05 in discovery set, validation set, early stage and late stage tumors).

|  | MSigDB ID of gene set: brief description | P value in blood (n=63) | P value in non-involved lung (n=50) | P value in discovery set (n=151) | P value in validation set (n=150) | P value in early stage tumors (n=246) | P value in late stage tumors (n=25) | P value in all tumors (n=301) |
| --- | --- | --- | --- | --- | --- | --- | --- | --- |
| 1 | TRANSMISSION_OF_NERVE_IMPULSE: Genes annotated by the GO term GO:0019226. The sequential electrochemical polarization and depolarization that travels across the membrane of a nerve cell (neuron) in response to stimulation. | 0.73 | 0.070 | 0.00010 | 0.0035 | 0.00045 | 0.00045 | <0.00005 |
| 2 | NEUROLOGICAL_SYSTEM_PROCESS: Genes annotated by the GO term GO:0050877. The processes pertaining to the functions of the nervous system of an organism. | 0.35 | 0.20 | 0.00020 | 0.0015 | 0.00015 | 0.00045 | <0.00005 |
| 3 | SYNAPTIC_TRANSMISSION: Genes annotated by the GO term GO:0007268. The process of communication from a neuron to a target (neuron, muscle, or secretory cell) across a synapse. | 0.56 | 0.040 | <0.00005 | 0.0011 | 0.00005 | 0.00025 | <0.00005 |
| 4 | AGUIRRE_PANCREAS_CHR9: Genes on chromosome 9 with copy-number-driven expression in pancreatic adenocarcinoma. | 0.25 | 0.57 | <0.00005 | 0.000050 | <0.00005 | 0.00004 | <0.00005 |
| 5 | HSA04080_NEUROACTIVE_LIGAND_RECEPTOR  _INTERACTION: Genes involved in neuroactive ligand-receptor interaction | 0.061 | 0.00050 | 0.00070 | <0.00005 | <0.00005 | 0.0042 | 0.000050 |
| 6 | BOQUEST_CD31PLUS_VS_CD31MINUS_DN: Genes overexpressed 3-fold or more in freshly isolated CD31- versus freshly isolated CD31+ cells | 0.25 | 0.068 | 0.00025 | 0.00015 | 0.00015 | 0.0020 | 0.000050 |
| 7 | NERVOUS_SYSTEM_DEVELOPMENT: Genes annotated by the GO term GO:0007399. The process whose specific outcome is the progression of nervous tissue over time, from its formation to its mature state. | 0.091 | 0.13 | 0.000050 | 0.015 | 0.0001 | 0.014 | 0.00020 |
| 8 | SYSTEM_PROCESS: Genes annotated by the GO term GO:0003008. A biological process, occurring at the level of an organ system pertinent to the function of the organism. An organ system is a regularly interacting or interdependent group of organs or tissues that work together to carry out a given biological process. | 0.18 | 0.15 | 0.0018 | 0.0045 | 0.0032 | 0.0043 | 0.00045 |
| 9 | CELL_CELL_SIGNALING: Genes annotated by the GO term GO:0007267. Any process that mediates the transfer of information from one cell to another. | 0.80 | 0.061 | 0.0015 | 0.013 | 0.0017 | 0.0030 | 0.00070 |
| 10 | HSA04140_REGULATION_OF_AUTOPHAGY: Genes involved in regulation of autophagy | 0.16 | <0.00005 | 0.00065 | 0.0046 | 0.00095 | 0.00010 | 0.00085 |
| 11 | SIGNAL_TRANSDUCTION: Genes annotated by the GO term GO:0007165. The cascade of processes by which a signal interacts with a receptor, causing a change in the level or activity of a second messenger or other downstream target, and ultimately effecting a change in the functioning of the cell. | 0.21 | 0.23 | 0.012 | 0.0019 | 0.011 | 0.022 | 0.0015 |
| 12 | KANG_TERT_DN: Expressed gene profile of ATSCs and ATSC-TERT cells and partial list of genes that were downregulated in ATSC-TERT cells | 0.023 | 0.16 | 0.00030 | 0.028 | 0.0084 | 0.0091 | 0.0015 |
| 13 | SYSTEM_DEVELOPMENT: Genes annotated by the GO term GO:0048731. The process whose specific outcome is the progression of an organismal system over time, from its formation to the mature structure. A system is a regularly interacting or interdependent group of organs or tissues that work together to carry out a given biological process. | 0.17 | 0.052 | 0.00085 | 0.017 | 0.0046 | 0.049 | 0.0025 |
| 14 | CELL_SURFACE_RECEPTOR_LINKED_SIGNAL  _TRANSDUCTION_GO_0007166: Genes annotated by the GO term GO:0007166. Any series of molecular signals initiated by the binding of an extracellular ligand to a receptor on the surface of the target cell. | 0.35 | 0.034 | 0.021 | 0.0017 | 0.027 | 0.00075 | 0.0031 |
| 15 | VENTRICLES_UP: Upregulated in the ventricles of healthy hearts, compared to atria | 0.37 | 0.55 | 0.0011 | 0.030 | 0.0073 | 0.0044 | 0.0035 |
| 16 | CELL_CELL_ADHESION: Genes annotated by the GO term GO:0016337. The attachment of one cell to another cell via adhesion molecules. | 0.53 | 0.069 | 0.0071 | 0.0085 | 0.0028 | 0.00035 | 0.0035 |
| 17 | MULTICELLULAR_ORGANISMAL_DEVELOPMENT: Genes annotated by the GO term GO:0007275. The biological process whose specific outcome is the progression of an organism over time from an initial condition (e.g. a zygote or a young adult) to a later condition (e.g. a multicellular animal or an aged adult). | 0.14 | 0.020 | 0.0021 | 0.016 | 0.012 | 0.035 | 0.0043 |
| 18 | HDACI_COLON_CUR24HRS_DN: Downregulated by curcumin at 24 hrs in SW260 colon carcinoma cells | 0.92 | <0.00005 | 0.0070 | 0.0020 | 0.0094 | 0.037 | 0.0050 |
| 19 | REGULATION_OF_MITOTIC_CELL_CYCLE: Genes annotated by the GO term GO:0007346. Any process that modulates the rate or extent of progress through the mitotic cell cycle. | 0.64 | <0.00005 | 0.0010 | 0.049 | 0.0075 | 0.017 | 0.0053 |
| 20 | SERUM_FIBROBLAST_CORE_DN: Core group of genes consistently down-regulated following exposure to serum in a variety of human fibroblast cell lines (higher expression in quiescent cells, not cell-cycle dependent) | 0.55 | 0.85 | 0.0083 | 0.013 | 0.016 | 0.0077 | 0.0053 |
| 21 | OLDONLY_FIBRO_DN: Downregulated in fibroblasts from old (but not Werner) individuals, compared to young | 0.26 | <0.00005 | 0.031 | 0.0030 | 0.020 | 0.047 | 0.0089 |
| 22 | CENTRAL_NERVOUS_SYSTEM_DEVELOPMENT: Genes annotated by the GO term GO:0007417. The process whose specific outcome is the progression of the central nervous system over time, from its formation to the mature structure. The central nervous system is the core nervous system that serves an integrating and coordinating function. In vertebrates it consists of the brain, spinal cord and spinal nerves. In those invertebrates with a central nervous system it typically consists of a brain, cerebral ganglia and a nerve cord. | 0.0015 | 0.085 | 0.0065 | 0.032 | 0.0048 | 0.035 | 0.0099 |
| 23 | NOVA2_KO_SPLICING: Genes that are alternatively spliced in the neocortex of mice deficient in the neuron-specific splicing factor Nova2, compared to wild-type controls | 0.28 | 0.11 | 0.0041 | 0.024 | 0.0035 | 0.019 | 0.011 |
| 24 | NEGATIVE_REGULATION_OF_CATALYTIC_ACTIVITY: Genes annotated by the GO term GO:0043086. Any process that stops or reduces the activity of an enzyme. | 0.43 | 0.62 | 0.0093 | 0.024 | 0.023 | 0.014 | 0.015 |
| 25 | GROWTH: Genes annotated by the GO term GO:0040007. The increase in size or mass of an entire organism, a part of an organism or a cell. | 0.42 | 0.86 | 0.034 | 0.0077 | 0.024 | 0.0027 | 0.018 |
| 26 | CHROMATIN_MODIFICATION: Genes annotated by the GO term GO:0016568. The alteration of DNA or protein in chromatin, which may result in changing the chromatin structure. | 0.98 | 0.71 | 0.019 | 0.022 | 0.049 | 0.0095 | 0.021 |
| 27 | CMV_HCMV_TIMECOURSE_ALL_DN: Down-regulated in fibroblasts following infection with human cytomegalovirus (at least 3-fold, with Affymetrix change call, in at least two consectutive timepoints) | 0.86 | 0.97 | 0.017 | 0.039 | 0.027 | 0.021 | 0.030 |
